# Oxidative Stress Implications in the Affective Disorders: Main Biomarkers, Animal Models Relevance, Genetic Perspectives, and Antioxidant Approaches

**DOI:** 10.1155/2016/3975101

**Published:** 2016-08-01

**Authors:** Ioana Miruna Balmus, Alin Ciobica, Iulia Antioch, Romeo Dobrin, Daniel Timofte

**Affiliations:** ^1^Department of Molecular and Experimental Biology, “Alexandru Ioan Cuza” University, 11 Carol I, 700506 Iaşi, Romania; ^2^Romanian Academy, Iasi Branch, 8 Carol I, 700505 Iaşi, Romania; ^3^“Grigore T. Popa” University of Medicine and Pharmacy, 16 Universitatii Street, 700115 Iasi, Romania

## Abstract

The correlation between the affective disorders and the almost ubiquitous pathological oxidative stress can be described in a multifactorial way, as an important mechanism of central nervous system impairment. Whether the obvious changes which occur in oxidative balance of the affective disorders are a part of the constitutive mechanism or a collateral effect yet remains as an interesting question. However it is now clear that oxidative stress is a component of these disorders, being characterized by different aspects in a disease-dependent manner. Still, there are a lot of controversies regarding the relevance of the oxidative stress status in most of the affective disorders and despite the fact that most of the studies are showing that the affective disorders development can be correlated to increased oxidative levels, there are various studies stating that oxidative stress is not linked with the mood changing tendencies. Thus, in this minireview we decided to describe the way in which oxidative stress is involved in the affective disorders development, by focusing on the main oxidative stress markers that could be used mechanistically and therapeutically in these deficiencies, the genetic perspectives, some antioxidant approaches, and the relevance of some animal models studies in this context.

## 1. Introduction

In the past few decades, a strong link between the inflammatory, oxidant, mitochondrial, and apoptotic markers versus the cognitive decline has been developed and theorized [1]. It seems that all these pathological background features are somehow molecularly linked, leading to a complex interaction between the cellular/molecular control and causes-effects conditioning. This is why, generally speaking, most of the neuropsychiatric disorders causes that are leading to the known and seen symptoms are a rather problematic matter to determine and to successfully discriminate from other collateral features. In this way, the neuropsychiatric diseases still remain partly unknown due to a multifactorial background. This may be the reason why no efficient specific treatment has been yet developed, the therapy relying only on symptomatic alleviation [2–4].

**Figure 1:**
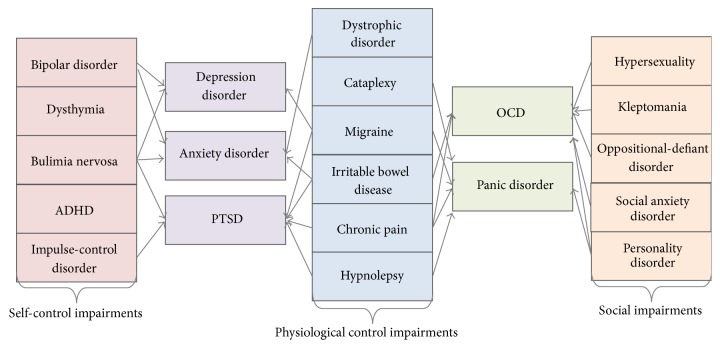
Affective disorders vertical classification (ADHD: attention deficit and hyperactivity disorder; PTSD: posttraumatic stress disorder; OCD: obsessive-compulsive disorder). Some of the symptoms for the affective disorders are quite distinct between the affective variants groups, while the main affective disorders (ANX, DD, PTSD, OSD, and PD) are more likely symptom combinations of the groups. Therefore, ANX, MDD, and PTSD exhibit both self-control discrepancies, as observed in bulimia, impulse-control impairment, or attention deficits, and physiological control alterations, such as irritable bowel disease, frequent migraines, or chronic pain. Furthermore, on the opposite side stand OCD and PD, which exhibit mainly social impairments, such as oppositional-defiant behaviour, social anxiety, and different personality discrepancies, as well as physiological impairments. In this way, it seems that the major affective syndromes can be classified given the general symptomatology tendencies in two groups: self-control-associated syndromes (DD, ANX, and PTSD) and social-hurdle syndromes (OCD, PD) (based on [6]).

This is also the case for the affective disorders or mood disorders, which are a group of well-studied related psychiatric disorders which have common socioaffective features and can accompany unipolar, bipolar, or schizoaffective syndromes [5]. The main spectrum is constituted of several psychiatric pathological conditions which occur in different combinations determining variable social or affective behaviour classified as mood impairments. The mainly known affective disorders are depressive disorder (DD), anxiety disorder (ANX), obsessive-compulsive disease (OCD), panic disorder (PD), and posttraumatic stress disorder (PTSD). Obviously, these pathological behaviours can gain different shades (Figure 1) in developing other affective variants such as self-control impairments, physiological control impairments, and social impairments [6].

Also, it seems that several cellular and molecular features of the affective disorders are quite similar, disregarding the specific clinical symptomatology. In this way, one of these aspects is oxidative stress status, which seems to be implicated in most of the different affective disorders, since it has been shown that increased oxidative damage occurs quite often in depression [7–9], anxiety [9, 10], bipolar disorder (BD) [10–14], panic disorder (PD) [15, 16], and also in obsessive-compulsive disorder [17].

Oxidative stress can be easily defined as the condition arising from the imbalance between toxic reactive oxygen species (ROS) and the antioxidant systems [1]. Shortly, the most studied ROS are the superoxide anion (O^2−^), hydroxyl radical (HO^−^), hydrogen peroxide (H_2_O_2_), nitric oxide (NO), peroxyl (ROO^−^), and reactive aldehyde (ROCH), while on the other side these reactive species are dealt with by the body in several ways, including the usage of the antioxidant enzymes (e.g., superoxide dismutase, SOD, that catalyzes the conversion of superoxide radicals to hydrogen peroxide, which is then converted into water by glutathione peroxidase, GPX, and catalase, CAT), as our group previously demonstrated on different occasions through most of the neuropsychiatric disorders [18–25].

Moreover, various tissues have different susceptibilities to oxidative stress. In fact, the correlation between the oxidative stress status and affective disorders development could arise from the vulnerability of central nervous system (CNS) to oxidative damage. Oxygen related free radicals and reactive species are both produced by the body, primarily as the result of the aerobic metabolism [26]. In the intra- and extraneuronal environment, these molecules have also important functions, such as synaptic plasticity and memory regulation [27]. More than that, the CNS tissues are rich in lipid molecules which are an easy target for oxidation reactions of the pathways in which they are involved [28]. In addition, the metabolism of some neurotransmitters is also based on redox potential transmission [29].

In this way, it has been shown, for example, that neuroinflammatory pathways activation together with CNS oxidative and nitrosative stress could play an important role in the DD's pathophysiological background [30]. Interestingly, it seems that ROS and RNS can cause immune response aberrations alongside molecular membranes structural alteration leading to immunogenic properties that could alert the immune system in the presence of oxidized fatty acids. This would be pathway through which DNA, proteins, lipids, and mitochondria damages can lead to the dysfunctions observed in DD [31].

Broad mitochondrial dysfunctions have also been reported in the context of ROS and RNS overproduction described in BD. As mitochondria are the most active organelles in ROS/RNS production, it seems that they also contribute to affective disorders pathological mechanisms. It has been shown that, in BD, the occurrence of mitochondrial DNA mutations and the occurrence of other mitochondrial diseases are rather high [32]. This could be the reason why mitochondrial metabolism correction could actually result in some alleviation of BD symptomatology [33].

In addition, a strong link between oxidative stress and anxiety-related phenotypes has been observed [34]. In this way, using genetically ANX-predisposed mice strains, the correlation between several antioxidant enzymes such as glutathione reductase-1 or glyoxalase-1 hyperactivity and intense anxiety behaviour has been described. Since then, many of the studies showed that ANX is a GABAergic and serotonergic modulated condition (as reviewed by [35]). Thus, it seems that both GABA and 5-HT are involved in the modulation of anxiety responses in the brain. Regarding the GABA release modulation, it seems that in anxiety the most important aspect is the 5-HT capacity to modulate the excitability of GABAergic interneurons. Several detailed studies on hippocampal response to serotonin stimulation via dorsal raphe fibers showed that serotonin specifically targets a subset of hippocampal interneurons involved in GABA B-mediated feed forward inhibition [36]. Moreover, GABA-A receptor agonists were showed to inhibit untrained anxiety reactions. In this way, intrahippocampal infusions of glutamatergic, serotonergic, and cholinergic compounds are thought to produce reliable antianxiety effects [37].

Also, GABA release modulation has been observed in other brain areas involved in anxiety behaviour regulation, such as dentate gyrus, which plays important roles in generating contextual memories of fear; entorhinal cortex, which modulates contextual fear memory extinction; piriform cortex which consists in amygdala, the fear center, frontal cortex involved in subcortical fear response, and anterior cingulate cortex-remote contextual fear memory [38].

Furthermore, a clear connection between fear response and oxidative damage was established being given that glyoxalase 1 cytotoxic substrate, methylglyoxal, is one of the well-known GABA agonists and also a potent modulator in oxidative stress and apoptosis [39]. In this way, Hassan et al. [40] demonstrated a close interaction between brain antioxidant system genes expression (glutathione reductase 1 and glyoxalase 1) and anxiety-like behaviour. Furthermore, the link between oxidative stress and emotional stress, such as fear and phobia, is based on the oxidative impairment caused in the brain by the imperfect gene expression that leads to oxidative and enzymatic unbalance. However the modulation properties of ROS on glutathione reductase 1 and glyoxalase 1 genes expression are yet unclear, as Hassan et al. [40] based their conclusions on lentiviral-modulated gene expression. The fact that antioxidant enzymes' genes overexpression was observed in fear-controlling brain areas in the absence of oxidative stimuli leads to the conclusion that there might be other stress-controlled mechanisms that are involved in anxiety-like behaviour occurrence.

Moreover, Hassan et al. [40] showed that oxidative stress may actually be the leading cause of ANX, while Masood group [41] found that glutathione synthesis inhibition may induce hippocampal and amygdala oxidative stress, leading to the assumption that hippocampal oxidative stress and ANX could be indeed connected.

In PD context, the latest studies also describe a strong connection between antioxidant enzymes activity and panic symptomatology [42, 43]. For instance, a certain phenotypic formula in antioxidant defense may be associated with gender specific PD development. Thus, the Pro198Leu polymorphism of the glutathione peroxidase 1 gene seems to participate in the development of anxiety-like behavioural phenotypes. Also, PD was newly characterized as an anxiety spectrum disorder due to the similarities between the oxidative mechanisms [44].

In the same way, it seems that mitochondrial dysfunction could be an important mechanism for the pathology of OCD [45]. Moreover, a correlation between mitochondrial disorders and oxidative stress in OCD was revealed due to a genetic variability of manganese-dependent superoxide dismutase and a small mitochondrial protein [46]. More than that, an oxidative imbalance has been reported in OCD patients, but unfortunately the exact pathway in which oxidative stress is implicated in OCD is still partially unclear [17].

The exact correlation between MDD and oxidative stress is also a major concern, since a close connection between glutamatergic hyperactivity and depression has been postulated [47]. Glutamate is the predominant excitatory neurotransmitter being responsible for synaptic plasticity, learning, memory, and locomotion. The glutamatergic system naturally regulates the glutamate concentrations in the synaptic shaft via both neuronal and glial receptors. Several stressful stimuli lead to excessive release of glutamate into the synapse that can cause glutamatergic hyperactivity, neurotoxicity, and cell death when neuronal receptors extendedly activated. Following excessive glutamate release, a decrease in brain GABA is observed since glutamate is being used by the brain to synthetize GABA [48]. Standing several brain imaging studies are also supporting this hypothesis, which showed that acute depression is associated with low prefrontal and occipital cortex GABA concentrations [49].

Thus, alongside presynaptic downregulation of GABAergic system, and therefore GABAergic neurons activity reduction, GABA-A receptor function may be impaired [50]. Furthermore, GABA(A) mediated neuronal inhibition induced by pre- and postsynaptic sites interaction with ROS may also contribute to the development of neuronal damage leading to neurotransmission impairments [51]. Also, several studies suggest that the glutamatergic system dysfunction is obvious due to the exceptional efficiency of ketamine in MDD treatment.

More specific studies on this aspect showed that increased NMDA receptor activity and glutamatergic synapse impairment are leading to depressive behaviour when localized in the prefrontal cortex. In this way, several studies managed to demonstrate equally hyperactive NMDA receptors and glutamatergic synapses both in depressive patients and in animal models of depressive behaviour [52, 53]. Thus, increased glutamate levels could lead to free radical formation by xanthine oxidase, for example, and further production of oxygen radicals and oxidative neurological damage. Also, nitric oxide-related toxicity caused by peroxynitrite formation in NO and superoxide anions reaction results in microfilament tyrosine residues nitration [54]. At the same time, Mg-SOD activity, which is positively modulated by ROS accumulation, could inhibit the mitochondrial respiratory chain and the glutamate transporter and therefore lead to glutamate-induced neurotoxicity [55].

However, MDD has been characterized as a progressive stage-related process of neurodegeneration caused by apoptosis, reduced neurogenesis or neuronal plasticity, and increased autoimmune responses. Thus, increased oxidative stress markers and neuroinflammation have been reported in the blood of depressed patients [56], but the molecular pathways through which impaired redox homeostasis interacts with the immune-inflammatory system in relation to MDD are still not clear.

Of course, there are still a lot of controversies regarding the relevance of the oxidative stress status in most of the affective disorders and despite the fact that most of the studies are showing that the affective disorders development can be correlated to increased oxidative levels (as we showed for most of the shortly described studies above), there are also some distinct studies reporting that oxidative stress may not be linked in any way, for example, with PTSD [57, 58].

Thus, in this minireview we decided to describe the way in which oxidative stress is involved in the affective disorders development, by focusing on the main oxidative stress markers that could be used mechanistically and therapeutically in these deficiencies, genetic perspectives, and antioxidant approaches, as well as the relevance of some animal models studies in this context.

## 2. Methodology

The information gathered for this review was searched in the main available databases (e.g., ScienceDirect, PubMed/Medline, Embase, and Google Scholar) taking into consideration just the articles in the English language. The selection process was conducted regardless of the articles publication date and included articles up to March 2016. Firstly, the publications were screened by title, then by abstract content, and then by full content. This inquiry was conducted by three separate researchers (Ioana Miruna Balmus, Alin Ciobica, and Iulia Antioch). Any differences of opinions were solution by common consent.

## 3. The Relevance of Some Oxidative Stress Markers and Their Mechanisms in the Affective Disorders

Currently, many hypotheses are describing the affective impairments. Depression development, for example, relies on psychological, psychosocial, hereditary, evolutionary, and biological combined factors. In this way, most of the theories rely on the monoamine hypothesis which states that serotonin, norepinephrine, and dopamine can assist the development of depression in a concentration-dependent manner. Thus, serotonin is thought to modulate other neurotransmitter systems and therefore any changes in its concentration may lead to unusual or aberrant neurotransmission [58]. Low serotonin levels are promoting low norepinephrine levels which could lead to DD [111]. Correlated to this, a widely known hypothesis stated that certain monoamine neurotransmitters can lead to affective impairments: norepinephrine deficits may be related to alertness and energy loss leading to anxiety, attention deficits, and loss of interest in life, while serotonin deficits is related to anxiety, obsessions, compulsions, and dopamine system impairment to attention and motivation loss [112]. Still, many limitations of the monoamine hypothesis led to the conclusion that DD may be rather complex almost certainly multifactorial [113, 114]. In this way oxidative stress and its main markers could represent some viable solution for the understanding and for a better management of some affective disorders.

In fact, clinical trials were the primary source of evidence that oxidative stress could be implicated in the pathogenesis of the affective disorders. In this way, it was shown that the mood stabilizers and antidepressant therapies possess high antioxidant potential [115]. Also, other studies showed that some oxidative stress markers are normalizing during or after the specific therapy applied for the affective episodes, suggesting that antidepressants could actually reduce oxidative stress levels [116]. In this way, it was actually shown that several antidepressants such as tianeptine [117], escitalopram [118], venlafaxine [119], or mirtazapine [120] could exert antioxidant effects.

However, there are still a lot of controversies about this subject, since some authors such as Bilici et al. showed that the administration of some selective serotonin reuptake inhibitors (SSRIs) for 3 months is generating a normalization in levels for some oxidative stress markers such as some antioxidative enzyme activities and lipid markers [121] or the Gałecki group, which demonstrated that fluoxetine given together with acetylsalicylic acid is decreasing the oxidative stress levels in patients with major depression disorder (MDD) [122], while on the other side the group of Sarandol stated no significant modifications in the oxidative stress levels after venlafaxine and sertraline administration for 6 weeks and/or the same Gałecki research group, which showed no modifications at all in the levels of some oxidative stress markers such as the glutathione peroxidase after 3 months of fluoxetine treatment [123, 124]. In this way, one possible explanation for this lack of homogenous results could be represented also by the dosage of antidepressant used, since, for example, 40 mg of fluoxetine could exert some antioxidant effects [125], which are not visible in other studies that used 10 or 20 mg, as in the aforementioned Gałecki et al. study [126] (as also described in [19]).

In fact, in MDD, while most of the authors have generally described decreased levels of GPX and increased levels of malondialdehyde (MDA), as a lipid peroxidation marker [123, 126], there are also controversies regarding the specific activity of some antioxidant enzymes such as SOD, which was reported to be decreased in patients with MDD [56], showing no significant modifications when compared to controls [127] or a significant increase in most of the studies [123, 124, 128, 129].

As we also mentioned before when we described the levels of SOD in some neuropsychiatric disorders [19, 130] this could be perhaps explained by the fact that SOD represents the first enzyme to get in contact with the free radicals and its increase may suggest some compensatory actions. However, when the specific activity of both SOD and GPX us decreasing, this will lead to an accumulation of hydrogen peroxide that will stimulate in a cascade of the lipid peroxidation processes and protein oxidation, which could explain some pathological manifestations observed in these disorders.

In this way, it seems that lipid peroxidation is an important component of the oxidative stress status observed in depression [131]. In fact, our group showed that subclassifying depression into different stages, based on chronicity (e.g., first episode versus recurrent depression), can actually predict significant differences in the levels of some lipid peroxidation markers such as MDA and also in the specific activity of the main antioxidant enzymes such as SOD and GPX [19]. Thus, perhaps an increased production of oxygen and nitrogen reactive species in these patients could generate a rapid consumption of the plasmatic antioxidants. Thus, in a so-called vicious cycle in the various staging of these affective pathologies, we could face an inadequate antioxidant enzymatic activity incapable of counteracting increased concentrations of free radicals and inflammatory processes, as we will show immediately. Moreover, similar facts were showed by our group in the case of the mild cognitive impairment and AD patients [18], so the aforementioned aspects could represent perhaps an important pathway in the development of these neuropsychiatric disorders.

Coming back to MDD, Dimopoulos et al. group [132] showed that plasma levels of isoprostane-8-epi-prostaglandin F2 alpha gets unusually high in elder patients with depressive symptoms. Moreover, Müller et al. [133] propose a new marker of oxidative stress, based on the fact that the brain membrane lipids are very important in depressive and anxiety disorders progression. In this way, it seems that low polyunsaturated fatty acids (PUFA) levels can be correlated with low antioxidant protection and an increased n-3 PUFA supply may reduce mood-related behaviours [133]. In fact, it seems that omega-3 fatty acids may actually alleviate some depression-related effects [134]. In this way, as we will insist on the last chapter of this review, dedicated to the possible antioxidant therapeutic approaches in most of the affective disorders treatment, several recent studies also showed that eicosapentaenoic acid supplementation was actually quite effective against primary depression [135]. Moreover, it has been also observed that GPX homologues could exert some antidepressant effects [136, 137], while Brown et al. [138] demonstrated that lipid peroxidation, DNA/RNA damage, and nitric oxide levels could be relevant markers in the MDD pathology.

As mentioned, several recent studies such as the one of Black et al. in 2015 [8] revealed that both 8-hydroxy-2-deoxyguanosine (8-OHdG) and F2-isoprostanes are increased in depression, suggesting a strong implication of inflammation and oxidative stress in its pathological mechanisms. Moreover, this recent finding supports the hypothesis that increased metabolic stress is present in depression contributing to its high somatic morbidity and mortality. In fact, there are opinions in the literature that MDD could be considered an inflammatory disorder, as judged mainly by the increased levels of the proinflammatory cytokines, such as interleukin-1b, interleukin-6, or tumor necrosis factor-alpha [139]. There is also a “vicious cycle” of pathogenic manifestations in this case, considering that depression could be correlated to an increased production of proinflammatory cytokines that, in turn, would lead to increased oxidative stress [140, 141], while also decreased levels of antioxidants/antioxidant enzymes could generate increased inflammatory response [142].

Brain antioxidant deficiencies also contribute to an oxidative damage which was observed in BD. In this way, glutathione was found in low concentrations in the prefrontal regions of bipolar patients [143], while downregulations of important antioxidant enzymes (such as superoxide dismutase 1, glutathione peroxidase 4, and glutathione S-transferase) were observed in hippocampus [144]. Moreover, considering that the thiobarbituric reactive substances (peroxidized species of lipids or lipid complexes) can easily change protein conformations and therefore disturbing lipid messengers signalling systems [145, 146], some authors found that, in BD, the oxidative stress to lipid structures could actually increase in a stage-dependent manner, disregarding the mood episode [147, 148]. On the other hand, as the nitric oxide is involved in the excessive release of glutamate and abnormal reactions to thiol proteic groups [26], it seems that the role of glutamate-induced oxidative stress via nitric oxide could be also extremely relevant in BD [148].

In addition, groups such as the one lead by Grande et al. [149] or Vieta et al. [150] suggested that alongside the progression of the BD, several markers such as neurotrophins and inflammatory cytokines (tumor necrosis factor-alpha (TNFa)) could be well correlated to the pathological evolution of the disorder. Moreover, Kapczinski et al. [151] stated even from 2009 that TNFa levels could represent an important marker in the bipolar disorder staging.

Also, it was stated that the lipid peroxidation processes could represent an important biomarker in BD progression, together with 8-OHdG, which can cause improper translation and protein aggregation [152] and with 5-hydroxymethylcytosine (5-HmC) [153].

Moreover, it seems that oxidative stress can alter brain activity through similar mechanisms, but with different visible behavioural manifestations. In this way, glyoxalase 1 is an enzyme which protects against carbonyl stress, reaction controlled by glutathione as a cofactor for this enzyme [154]. On the ground that glutathione reductase 1 and glyoxalase 1 are antioxidant factors which are highly correlated to ANX behaviour, many studies have been conducted in order to find the nature of this correlation. Thus, Hovatta et al. [33] showed that overexpression of the glutathione reductase 1 and glyoxalase 1 gene leads to anxiety-like behaviours, while inhibition of glyoxalase 1 expression produces only low intensity anxiety-like behaviours. Also, based on the fact that excessive ROS accumulation induces overexpression of these genes and therefore intense activity of the enzymes, it can be speculated that they could regulate ANX. However, there are also controversial results in this area of research, since these findings were discordant with other studies which showed that glyoxalase 1 may be a marker for the trait anxiety [155, 156].

Also, mechanistically speaking anxiety could be related to low levels of gamma-aminobutyric acid (GABA) occurrence which is reducing brain activity [157]. In this way, either overactivation or underinhibition can lead to cortical and limbic glutamate neurotransmission through N-methyl-D-aspartate (NMDA) receptors that is linked to an excess of stimulatory glutamate, calcium influx, or insufficient GABA or GABA receptor function deficits. Additionally, the research on the GABAergic system has been performed on PD and OCD animal models and patients [158, 159], demonstrating also that oxidative metabolism can affect the regulation of ANX behaviour. In this way, it has been shown that in oxidant conditions and due to the lipid-rich constitution of brain, lipid peroxidation increases which causes membrane fluidity impairment and probably impairments in receptors, enzymes, and ion channels functions [160]. Therefore, it is quite possible that oxidative stress could alter neurotransmission, cell signalling, and therefore brain activity in these pathologies [161].

## 4. Oxidative Stress Implications in Some Animal Models of Affective Disorders

To this date, the generation of various animal models is considered an extremely valuable tool in understanding the mechanisms behind a variety of specific diseases. Also, animal models are widely and efficiently used in the affective disorders research area, considering the obvious ethical constraints in using human subjects and the impossibility to control the human individual variability [144].

Of course, the animal models are not the perfect representation of the complex human diseases, especially considering the fact that psychiatric concepts such as self-esteem, recurrent thoughts of death [162], or fear of losing control [163] are not reproducible in this case. Instead, they are created to mimic certain characteristics of the disease or a behavioural dimension specific to that psychiatric pathology (e.g., affective disease in this case) [164].

**Figure 2:**
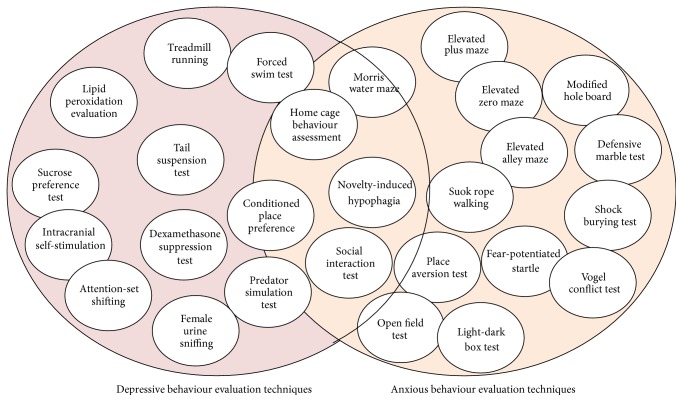
Behavioural tests battery used in depression/anxiety assessment [165]. Due to the fact that depressive and anxious behaviours are interconnected, and in some cases interdependent, it is very important for the difference of anxiety as a trait or as a symptom, for example, to be clearly defined. Thus, several evaluation techniques can only evaluate depressive behaviour (the tests shown in the left side of the picture), being useful in determining clear depressive traits. On the opposite side the typical anxious behaviour techniques stand which are meant to evaluate general and conditioned anxiety, while in the middle the depressive-related anxiety techniques stand, which can be used in both depression and anxiety evaluation. This aspect can be crucial when elaborating complex hypothesis regarding common symptomatology and behaviour which leads to elucidating information in affective syndromes etiology.

Due to this fact, it is extremely important to correctly assess the specific affective spectrum behaviour. In order to do this, there are many behavioural tests which can be successfully used. A more comprehensive example is presented in Figure 2, in regard to the various tests which can assess depressive and anxious behaviour.

Also, these animal models must fulfill several criteria to be validated. For this reason, they must be comparable to the human dysfunction in the aspects of symptomatology (face validity), treatment manners (predictive validity), similar causative neurobiological factors (construct validity), and common etiology (etiological validity) [59]. Another aspect that must be met is repeatability between laboratories and various studies [60].

Constantly, new models are designed or the existing ones are improved due to the need of a higher accuracy. It is also the case of affective disorders modelling in animals considering ethological aspects, genetics, surgical procedures, chemical induction, or their combination resulting in a multitude of animal models (Table 1).

In this way, it seems that oxidative impairments observed in the animal models of affective disorders are somehow similar to the disparities found in humans. Thus, Brocardo's team, for example, demonstrated the presence of increased levels of oxidative damage in a rat model of fetal alcohol exposure, in which they created the conditions of anxiety- and depression-like behaviour. They recorded significantly higher levels of lipid peroxidation and protein oxidation measured in the hippocampus and cerebellum, while physical exercising displayed protective effects in this matter and increased the rates of glutathione [104].

Also, it was showed that in BD animal models there are various alterations for the protein oxidation markers, with the specific activity of SOD and CAT being increased and GPX activity decreased. Also, the levels of lipid peroxidation markers were found to be increased, which correlated to low rates of glutathione and vitamin C. Moreover, the administration of lithium and valproate in these cases was associated with a significant reduction for the lipid peroxidation processes in the hippocampus and the prefrontal cortex [90–93].

In other several models of depression in rats it was showed that these animals exhibit alterations of some oxidative mechanisms in the form of glutathione levels depletion, decrease in GPX specific activity, lower levels of vitamin C, or increased rates of lipid peroxidation and nitric oxides [166, 167]. Another study also showed that lamotrigine, aripiprazole, and escitalopram exerted some protective effects against depression linked GPX, glutathione, and vitamin C deficiency and also decreased lipid peroxidation levels. Moreover, from the aforementioned three drugs, it seems that lamotrigine was associated with the strongest antioxidant protective abilities [168].

Also, we can mention here the study of Kumar group, which used an immobilization stress animal model of anxiety and proved that the six-hour time spent in restraint could considerably increase the brain concentrations of lipid peroxidation markers and nitrite in the animals. Furthermore, the same anxiety model had important effects on brain glutathione and catalase rates which were significantly decreased compared to the control group [105].

Another animal model of affective disorder induced by ouabain (African plant derived toxic substance) single intracerebroventricular injection which actually could mimic the conditions of mania and that is course characteristic to the bipolar disorder resulted in increased thiobarbituric acid reactive substances (TBARS) and carbonyl levels especially in the frontal cortex and hippocampus area, while elevated SOD activity and reduced CAT specific activity were also reported in the aforementioned central areas [95]. Moreover, the same group showed that sodium butyrate (e.g., with the inhibition of Na^+^/K^+^-ATPase produced by ouabain) could counteract the oxidative alterations induced by specific toxin administration, through the reversal of the protein and lipid disturbances found in the hippocampus and prefrontal cortex of injected rats and by increasing CAT specific activity [169].

In addition phenelzine, which is a monoamine oxidase inhibitor drug, showed great antioxidative defense potential, being capable of reducing the reactive oxygen species formation and the scavenging proprieties of hydrogen peroxide [170]. Also venlafaxine, a drug from the selective serotonin reuptake inhibitors group, was able to reverse the deficits in glutathione (GSH) rates and also to decrease the levels of hippocampal MDA and nitric oxide (NO) induced by the specific stress depression tests such as forced swim test and tail suspension [171].

In addition, the well-known antioxidant ascorbic acid [172] was reported to reverse oxidative damage in an induced model of depressive disorder, as compared to fluoxetine-treated controls, by mainly increasing the specific activity of CAT and glutathione reductase [108].

Oxidative unbalance was also demonstrated in anxiety models by mainly pointing out the presence of increased lipid peroxidation, protein carboxylation, and protein thiol oxidation and decreased vitamin E levels [109, 173].

Furthermore, other evidences of oxidative stress in an anxiety rat model of social stress were demonstrated by an important increase of plasma 8-isoprostane and hippocampus protein carbonylation, but interestingly without any changes in prefrontal cortex and amygdala regions [107].

Also, decreased antioxidant enzyme rates of Mn SOD and Cu/Zn SOD in the hippocampus were found in a modified model of resident-intruder paradigm to highlight social stress (e.g., social defeat model) [110].

In addition, employing a model of PTSD induced by a single prolonged stress, it was noticed that the decreased levels of glutathione reductase found in the amygdala significantly elevated when grape powder treatment was applied. Also, when grape powder was given before the inducing of PTSD model, it was observed that raises in oxidative rates and inflammation were prevented, as proven, for example, by the analysis of plasma 8-isoprostane levels [174].

In addition, by using a chronic social isolation model which is designed to induce depressive and anxiety-like behaviour in rats, some other authors studied the effect on the hepatic oxidative stress and inflammation levels for olanzapine, an atypical antipsychotic that is also used sometimes as an adjuvant in anxiety or depressive states of bipolar disorder [175]. In this way, they saw that although the drug was able to reverse decreased hepatic glutathione levels, it did not alter the elevated hepatic proinflammatory cytokines, possibly indicating that it might have favourable antioxidative proprieties, but no effect on inflammation [176]. In fact, it has been observed after studying the effects of treatments with typical and atypical antipsychotics that while the first ones presented mainly prooxidant effects [177–179], the latter has proven to have an important antioxidant capacity [180, 181]. Also, our group previously showed some important antioxidant modifications for some atypical antipsychotics such as quetiapine, olanzapine, and risperidone [130].

In fact, we also demonstrated the relevance of some animal studies affective manifestations, especially on anxiety-related behaviour in the elevated plus maze specific test and the correlation of its factors (e.g., time in open arms, head-dipping, and stretching behaviour) with the main markers of the oxidative stress from the amygdala (e.g., SOD, GPX, or MDA), as a result of angiotensin (1–7) or angiotensin II blockers administration, which resulted in anxiolytic effects [22, 182, 183].

In addition, a restoration for the lipid peroxidation processes and nitrite concentration was obtained after coadministration of melatonin and buspirone in a specific immobilization stress test known to induce anxiety-like behaviour [105]. Also, other antioxidant and anxiolytic agents have been proven to be effective against the oxidative stress status such as epigallocatechin gallate (EGCG), green tea polyphenol [184], and chlorogenic acid, a dietary polyphenol [185], as we will insist in the last chapter of this minireview dedicated to the relevance of the antioxidant administration in the affective disorders.

Therefore, oxidative stress metabolism appears to have important implications in the evolution of replicated affective disorders aspects in animal models [94], as synthesized in Table 2, but there is not yet a clear explanation to why these processes occur. Consequently, the need to further develop animal models and strategies is highlighted that will eventually lead to an elucidation of the oxidative stress mechanism in the affective disorders.

## 5. Genetic Aspects in the Understanding of Oxidative Stress Implications in Affective Disorders

The implications of the genetic status into the most common diseases have been lately intensely argued. Thus, in order to find an explanation for the high prevalence of several diseases, the paradigm of genetic inheritance has been discussed. In this way, it seems that several classical diseases such as high blood pressure, diabetes, and also some neurodegenerative disorders may be able to pass between generations [186]. In fact, it is now well accepted that a strong inheritable genetic component might be involved in the pathogenesis of the affective disorders [187].

In this way, many genes have been described to be involved in affective disorders' pathology. They may be associated with brain growth factors, signal molecules, receptors, chelation, and transport factors, while some of them are actually genes which encode several enzymes and factors implicated in brain oxidative status [187].

Although the way in which the genetic component actually increases the risk for affective disorders is not fully understood, it is believed that the genetic components of affective disorders may be a result of multiple gene modifications that lead to a specific environmental factor-dependent liability. In this way, the bipolar disorder was found to be the most likely to be inherited (up to 80% probability by additive genetic factors) [188], while major depression (40%–70%) [189] and anxiety (40–50%) [190] are less probable to be inherited by the descendants.

Therefore, a contradiction between theoretical heritability and susceptibility premises and the actual clinical status may occur. A good example in this way is the genetic component of PTSD, which seems to occur in particular genetic context and under particular environmental factors [191]. Moreover, a significant interaction between three polymorphisms in the GABA receptor gene was reported to be involved in PTSD prediction in correlation to childhood trauma severity [192]. Besides the neurotransmission regulatory function of GABA receptors, it seems that GABA alpha-2 receptor is also implicated in stress modulation via chloride cotransporters domains, which are also activated by oxidative stress responsive kinases [193]. In this way, since oxidative stress may be a molecular response to psychological stress, it might actually modulate the regulator of G-protein signalling 2 (RGS2) [194], which is a part of the adrenergic receptors during conditioned fear response. Also, recently several single nucleotide polymorphisms of the FK506 binding protein 5 (FKBP5) were found to interact with childhood trauma in order to create PTSD susceptibility [195].

Similarly, oxidative stress may be involved in FKBP5 functionality due to the interaction between FOXO1 (a transcription factor involved in cell survival and modulated by oxidative stress), glucocorticoid receptors, and increased levels of psychological stress [196].

In addition, the female predisposition to PTSD may be modulated by a recently found single nucleotide polymorphism of estrogen response element, found on pituitary adenylate-cyclase 1 receptor gene [197]. It seems that pituitary adenylate-cyclase activating peptide/pituitary adenylate-cyclase 1 pathway exhibits a role in psychological stress response, which is dependent on an estrogen response element that conveys sex specific-modulation of fear response. Moreover, this pathway seems to be involved also in an oxidative stress protective system against ROS-induced mitochondrial dysfunctions and apoptosis [198].

Also, dopamine and serotonin receptors polymorphisms may also be involved in PTSD predisposition due to the limbic-frontal neurocircuitry complexity. In this way, dopamine transporter SLC6A3 and promoter region of the serotonin transporter genes polymorphisms seem to give high risk of PTSD, especially in increased risk environment factors [192]. Dopamine receptor D2 association with oxidative stress is rather controversial, considering, for example, that one recent study correlated a dopamine D2 receptor antagonist and anti-Parkinson medication with reduced excitotoxicity and therefore reduced neuronal apoptosis in oxidative stress conditions [199].

Another previous study also associated D2 and D3 dopamine receptor agonists with glutamate oxidative stress inhibition in oxygen/glucose deprivation models [200]. In addition, other groups correlated organophosphates exposure with oxidative stress and alterations in brain dopamine and serotonin receptors of young rats, but still no actual correlation between oxidative stress and these receptors has been proposed [201].

In fact, although a clear correlation between genetic components and PTSD has been made and all of these genes may be directly or indirectly implicated in oxidative stress modulation or development, no previous correlation between all of them is available. Therefore, since the predisposition to PTSD through these genes polymorphisms has been shown, the oxidative stress pathways in which they may be involved are almost unknown in PTSD conditions.

In this way, it can be stated that the genetic component, the environmental risk factors, and their interaction in the affective disorders development context are rather variable. Based on this observation, the latest studies in PTSD genetics actually revealed that identifying the specific genes or neurobiological pathways involved in PTSD development and the specific modifiable environments associated with PTSD risk (as well as the mechanism of interaction between the two) could broaden posttrauma intervention approaches in PTSD therapy or even result in some prevention mechanism [191].

Modern molecular biology and developmental biology rely on a crucial paradigm. As all living organisms are the result of a complex interaction between genome and environment, the mental disorders seem not to deviate from this pattern. In this way, interesting questions could arise: in what way the genetic component would formulate a sufficient background for affective disorders pathological development and, on the other way around, how complex would the environmental interaction be in order to provide sufficient risk for pathologies to occur via oxidative stress development? Therefore it seems that both questions eventually got answers in the way that it has been shown by familial studies [202, 203] that several genetic modifications (mutations or polymorphisms) in key genes could give rise to a sufficient mood imbalance background.

Unfortunately, as the familial cases are thought to be easier to screen and to prevent, these are only 5 to 10% of all cases. On the other hand, twin and adoption studies [204, 205] revealed that a close interaction exists between the genetic background and the environment, raising several environmental risk factors which could play an important role in building up the risk for affective disorders development.

There are plenty of studies that revealed the genetic component implications in the affective disorders occurrence and rarely the same susceptibility locus shows up repeatedly. This is the reason why a genetic screening in affective disorders is hard to produce any preventive actions.

In this way, a variety of genes have been shown to be involved in affective disorders development (reviewed by [187]). Association analyses of PD genetics showed several classical candidates such as monoamine oxidase A (MAOA), catechol-O-methyl transferase (COMT), adenosine A2A receptor (ADORA2A), and cholecystokinin B receptor (CCK-BR) genes [205]. COMT gene, for example, codes for a catecholamine catabolic breakdown enzyme, which is known to be involved in anxiety development as high levels of COMT have been observed in patient's serum [206]. The implication of the most common COMT polymorphism in PD is also rather controversial due to extremely different study results (reviewed by [205]). In this way, although there are studies which negatively correlate COMT with PD, it seems that this polymorphism remains as one of the most consistent findings in PD genetics.

Furthermore, the correlation between COMT and oxidative stress has not been studied much. Still there are several studies which report high COMT activity and high oxidative stress levels in vitiligo patients [207, 208]; a more relevant correlation between genetic implications in PD and oxidative stress may be made regarding the mitochondrial monoamine oxidase.

Also, MAO has been correlated with PD due to several polymorphisms which modulate MAO gene transcriptional activity [209]. Furthermore, a gender specific modulation has been demonstrated and was associated with PD in several populations [210]. The association remains controversial due to the fact that in other populations this correlation failed to be shown [211].

Also, the cholecystokinin (CCK) neuropeptide has been associated with PD development. It seems that an interaction between CCK and dopamine may be involved in panic attacks modulation [212]. In fact, ambiguous results have been obtained through time in several studies and therefore the exact correlation between CCK and PD is not known, although it seems that CCK A receptor and dopamine D5 genes are closely situated on the short arm of the fourth chromosome [212].

Another interesting association is referring to connection between the serotonin transporter gene and both PD [213] and OCD [214] pathologies. This gene is coding for a protein affected by selective serotonin reuptake inhibitor (SSRI) medications, which are of course frequently used in anxiety disorders treatment [215]. Interestingly enough, discrimination was made by showing that the modifications that lead to PD or OCD are different and a question arises: why the way in which a molecule is modified can change the pathological features? Maybe the answer relies on the genetic components involved in these two diseases development. In this way, the same gene may possess different polymorphic alleles of which different or opposed interactions lead to different results. For example, the serotonin receptor 2A was associated with both PTSD and PD [216].

Regarding the BD genetic component, it seems that most of the genetic studies on this matter focused on the neurotransmitter systems, which can be influenced by medication, and particularly dopamine, serotonin, and noradrenalin systems. In this way, direct implications were demonstrated for the monoamine oxidase A, 5-hydroxytryptamine transporter, and catechol-O-methyltransferase genes [217–220]. Also, the implications of these molecules in oxidative stress status have been partially explained, but no direct correlative study has been carried out. Moreover, Menazza et al. [221] showed that MAOA activity may increase mitochondrial ROS production, which will lead to increased oxidative stress and myofiber damage. In this way, increased MAOA activity in brain tissue may also lead to increased oxidative stress, knowing that the neurons are mitochondria-rich high energy consumers. In the same way, COMT activity is thought to be oxidative stress promoter in association with high catalase activity in melanocytes and melanin biosynthesis [208].

Also, while COMT plays an important role in the brain catechol amines degradation, it also degrades dopamine in the prefrontal cortex area which leads to working memory correlated tasks resolving. Since impaired working memory has also been correlated to oxidative stress and damage [222], it might be possible that high COMT activity is associated with both agitation/disorientation and oxidative stress. Later, D-amino acid oxidase activator gene and brain derived neurotrophic factor gene became also of great interest, but it seems that no actual evidence was found in this matter [223, 224], mainly due to the fact that most of these genes are reported as schizophrenia susceptibility genes too. Surprisingly, it can be observed that both D-amino acid oxidase activator and brain derived neurotrophic factor are involved in several oxidative stress pathways [14, 225], but a direct correlation between these two and oxidative stress in BD has not been yet showed.

In the same way, several susceptibility genes were shown in DD and ANX development. In addition, the brain derived neurotrophic factor (BDNF) polymorphism Val66Met thought to be implicated in BD was also investigated in DD. Just as in other genes' case, the results were quite controversial. In this way, no significant association with BDNF polymorphism or inconsistent evidence was reported [226, 227]. In spite of these reports, other variations in the BDNF gene may be influencing the susceptibility to DD [228]. Thus, recently a link between BDNF and oxidative stress has been confirmed in schizophrenia [229]. It seems that the patients exhibit a significant decrease in BDNF levels and also in the activities of SOD and GPX. Moreover, Numakawa et al. [230] showed that significant correlations can be made regarding BDNF and SOD specific activity. Also, they suggested that an interaction between BDNF and CAT could be associated with the positive and negative syndrome scale (PANSS) as a cognitive factor. Furthermore, a similar PANSS factor (PANSS depressive factor) can be correlated with the interaction between BDNF and GPX. In this way, a possible association between BDNF and inflammatory cytokines and also hypothalamic-pituitary-adrenal (HPA) axis could emerge.

Another gene thought to be implicated in DD is tryptophan hydroxylase gene, which encodes for an important rate-limiting enzyme of brain serotonin synthesis [231]. It seems also that a specific brain isoform of tryptophan hydroxylase (TPH2) may be the connection between serotonergic systems and depression and BD [232]. In this way, both Zill et al. [232] and Zhang et al. [233] groups reported genetic modifications that could link TPH2 gene to DD susceptibility. Furthermore, Kuhn et al. [234] reported a possible implication of the oxidative stress status in TPH2 activity, whereas THP2 oxidation leads to low TPH2 enzymatic activity. In addition, it seems that 5-hydroxytryptamine (5HT) synthesis by miss folding and aggregation due to the cysteine-rich structure could also be highly susceptible to oxidative damage [235]. Furthermore, a very recent report showed increased systemic oxidative stress in TPH2 knock-out mice and also increased lipid metabolism impairments which might be implicated in serotonin deficiency [235].

Other genes were similarly correlated to the DD development, especially considering the wide implication in the oxidative stress status and the association between high oxidative stress and depressive symptoms. In this way, it was reported that a polymorphic variant glutamic acid decarboxylase 2 (GAD2) that is described as an enzyme involved in GABA synthesis and which seems to be severely impaired in ANX disorders [236] may be also involved in DD [237]. It also seems that GAD2 enzyme is involved in an extensive antioxidant system yielded by the astrocytes. In this way, several reports showed that enhanced GAD2 activity may contribute to neuronal protection from oxidative stress in vitro neuronal tissue cultures [238, 239].

Also, controversial results were obtained in the case of the polymorphisms for the regulator of G-protein signalling 2 (RGS2) gene, which could be implicated in anxiety-like behaviours [240], but also in PTSD, emotional distress, and PD [241, 242]. In fact, a correlation between RGS2 and oxidative stress was made in a report regarding the postischemic RGS2 upregulation, which leads to enhanced apoptosis in astrocytes via oxygen-glucose deprivation [243]. Similarly, another RGS family protein, called RGS4, has been correlated with oxidative stress in postischemic and neurodegenerative disorders. In this way, it seems that a common lipid peroxidation product such as 4-hydroxy-2-nonenal can inhibit RGS4, which further impairs the GTPase activity [244].

Several single nucleotide polymorphisms (SNPs) within the transcriptional coactivator PPARGC1A were also associated with the anxiety phenotypes. PPARGC1A was actually discovered in the muscle cells and brown fat and thought to stimulate mitochondrial biogenesis by increasing oxidative phosphorylation and by enhancing oxidative respiration [245], but it has been shortly connected to the nuclear respiratory factors 1 (NRF1) and 2 (NRF2), which are linked to oxidative stress and also well correlated to ANX both in human and rodent models [23, 38].

However, we have to mention that a strong correlation between genetics, oxidative stress, and affective disorders has not been made quite clear due to the extreme genetic variability of the individuals. Interestingly enough, many correlations were observed between these susceptibility loci and oxidative stress status observed in affective disorders. Also, as mentioned before, some of the genes code for proteins which are important enzymes involved in oxidative or phosphorylation reactions which commonly use ROS. In this way, at least hypothetically and theoretically, a link between the neuroprogression biomarkers and the decline observed in affective disorders can be made. Also, due to the complex mechanisms underlying the pathophysiology of these diseases, the way in which inflammatory processes, oxidative stress, mitochondrial dysfunctions, and apoptosis are interacting is rather problematic and controversial.

## 6. Antioxidant Approaches for the Affective Disorders Treatment

Of course, the reason behind studying the connection between the affective disorders and the oxidative stress status is represented by the need of exploring new approaches towards the management of these important psychiatric disorders. This acute need of finding new therapeutic methods is sustained by the position these disorders are occupying on a worldwide scale. In this way, according to an estimative perspective given by the World Health Organization (WHO), major depressive disorder will be the second health problem worldwide by the year of 2020 [246]. Even more, it seems that in Europe and USA anxiety disorders are the most prevalent psychiatric conditions [247–249].

In this way, by knowing that oxidative stress is an important component in many diseases, the idea of counteracting its effects emerged lately in the literature. Thus, several approaches were designed, such as oxidant potential inhibition and antioxidant system potentiation approaches.

Therefore several studies showed that oxidative stress in the affective disorders can also be inhibited by psychiatric disease therapy itself, as this was demonstrated to be a causative component [11, 250, 251]. For instance, lithium can exhibit mood stabilizing properties, but also antioxidant potential, as some early phase studies regarding the oxidative stress status in BD showed [252]. Thus, a six-week lithium therapy can successfully decrease lipid peroxidation markers and restore SOD plasma levels [253]. Moreover, these results were comparable to prior studies on BD patients after manic and euthymic episodes [124, 254]. Furthermore, Banerjee et al. [254] group showed that lithium antioxidant activity is linked to Na^+^-K^+^-ATPase activity.

Also, when the modification of oxidative stress markers was followed in patients with depressive disorder after fluoxetine administration for three months, it was found that the levels of some antioxidant enzymes like Cu/Zn-SOD and CAT are significantly higher in the patients group versus the controls [124]. Moreover, it seems that also MDA levels are significantly lower during MDD medication [255].

Additionally, it seems that oxidative unbalance in social phobia patients can be corrected after eight weeks of citalopram administration [256].

Moreover, the normalization of these parameters over the course of therapy has been demonstrated by a classical case report of twin brothers diagnosed with mania. In fact, this was presented as a situation where one brother accepted therapy and the TBARS or SOD levels came back to normal parameters after medication, whereas the other brother refused the medication and these markers remained unchanged, as well as the manic symptomatology [257].

Also, it was noticed that the aforementioned lithium therapy can affect SOD activity in the BD patients, but also in healthy volunteers exposed to this medication [258]. In addition, consistent evidences are showing that the inhibition of oxidative damaging processes provoked by injuries to rat cerebral cortical cells by the chronic treatment with lithium is remarkably efficient [259]. It also appears that lithium can boost GSH rates and also the expression of glutamate-cysteine ligase [31], while also helping antioxidant defense mechanisms through the management of GST [260]. Moreover, additional reports indicated that lithium therapy in rats can increase the specific activity of SOD and GPX in the brain [261]. Also several human subjects treated with lithium showed that decreased NO levels and increased SOD activity can occur, as compared to first and the 30th day of hospital admission [262]. In addition, in a 3-month clinical trial it was showed that increased GPX activity can be observed after lithium treatment, as compared to before treatment determinations and control subjects [126].

Anti-inflammatory and antioxidant properties were also showed for the antidepressants such as clomipramine and imipramine, which resulted in decreased NO levels [263]. In addition, antioxidant properties and decreased NO levels were reported for several SSRIs such as fluoxetine, citalopram, fluvoxamine, or sertraline, together with reduced xanthine oxidase activity [264] and lipid peroxidation processes [265].

However, there are still a lot of controversies regarding the effects for the specific medication used for the affective disorders on the oxidative stress status, since some other studies suggested that antidepressant therapies could actually be a facilitator factor for the oxidative stress generation. In this way fluoxetine (as a fluorinated product) can induce hepatotoxicity, as a result to increased oxidative stress activity [266]. Also, amitriptyline can exhibit prooxidant activity as a result to coenzyme Q10 life shortage and lipid peroxidation potentiation [267, 268]. More than that, oxidative stress evaluation through F2-isoprostanes quantification [269] in depressed patients treated with sertraline or bupropion for eight months revealed high rates of oxidation, despite some psychiatric improvements [139]. In this way, although several methodological issues were proposed, a clear argument of a possible oxidative stress potentiation due to antidepressant medication was not yet very clearly theorized [270].

In fact, in a specific review, Michel et al. group suggested that antidepressant therapy and modulation of oxidative stress are linked and are contributing in a specific way to the oxidative balance [7, 127]. Although, in the context of depression, various studies highlighted that GPX activity becomes normal subsequently to subchronic therapy with antidepressants [56] and NO levels substantially diminish [262], it can be noted that, in the treatment course with fluoxetine, the specifications of oxidative and antioxidant context did not modify flagrantly, while including acetylsalicylic acid in the treatment scheme resulted in a significant improvement for the oxidative stress status [124]. One important argument for this could be represented by the fact that the depressive episodes in human individuals are defined by the presence of inflammatory components, which are representing of course an originating site for the reactive oxygen species [7, 270, 271].

Consequently the inclusion of a nonsteroidal anti-inflammatory drug could counteract inflammation and therefore could exert antioxidant actions. Several data are adding up to the fundaments of this hypothesis which are emphasising on the intensified features for the association of antidepressant therapy with antioxidants such as omega-3 acid [271] and N-acetylcysteine [272] in depressed persons and also anxiety SSRI resistant adolescents, all of these resulting in a better outcome for the treatment course [11]. Again, this enhancement of antidepressant power phenomenon tested in anxiety SSRI resistant adolescent patients proved that the treatment association with N-acetylcysteine (NAC) resulted in anxiolytic effects [273].

As in the present paper we referred to the main antioxidant markers as being mainly enzymes, it must be stated of course that antioxidant defense is also constituted in a nonenzymatic component (e.g., ascorbic acid, *β*-carotene, melatonin, coenzyme Q10, vitamin E, zinc, and glutathione) [274]. In this way, for example, in MDD it is important to observe the nonenzymatic antioxidant defense dynamics in order to evaluate the psychiatric treatment yielding and to determine future mood attacks [275]. Furthermore, it seems that many biological active molecules are implicated in oxidative stress such as folic acid (vitamin B9) [276]. In fact, it has been proven that vitamin B9 supplementation can induce several important changes in the biochemical context of the monopolar and bipolar depression [277].

Coming back to NAC, which is a precursor of glutathione, it has been shown that it can significantly improve the standard therapy, as a double-blind randomized placebo-controlled clinical study performed by Ng et al. in 2008 demonstrated [13]. In fact, NAC is considered a product which can modulate glutathione levels [149] and it is already cited as an adjuvant in BD therapy [278]. In addition, some studies showed that NAC could improve OCD-related trichotillomania (pathological nail-biting and skin-picking) [279]. Still, there are also controversies regarding the usage of it in the affective disorders, since the same author conducted another study in order to evaluate the NAC potential to alleviate DD outbreaks, with no significant results obtained in regard to its antidepressant potential [280].

Also, melatonin seems to be implicated in the antioxidant defence, being in fact recognised as a ROS scavenger [281]. In fact, several studies correlated the sleep deprivation to mood disorders via melatonin secretion impairments [282]. In addition, melatonin modulators are being suggested as potent mood regulators in BD, when used as adjuvant therapy to valproate or lithium administration [283]. Moreover, considering the well-known implications of melatonin in regulation of the circadian rhythm, melatonergic products have been noticed to have antidepressant capacity in depression encountered in shift workers [282, 284].

In addition, copper is another essential element for the normal functioning of the antioxidant system, which also plays an important role in immune system modulation, myelin formation, erythropoiesis, and the synthesis of hormones [155, 156]. Also, changes in copper level were noted in depressed patients [285]. In this way, knowing that alterations in copper levels could lead to deficiencies related to anaemia, neuronal degeneration, and cardiac and immune dysfunctions or that the exciding levels of copper could cause cellular instability [286, 287] there is an obvious need to control its homeostasis [288]. Moreover, although the proof of its implications in the oxidative mechanisms is pretty solid, some animal models studies contradicted the clinical findings [285] and therefore, there is still a need for the determination of the real benefits for the copper therapy in patients with depression [288].

Also in the case of zinc, there are reports demonstrating that it could exert some antioxidant effects, with proven effectiveness in clinical and preclinical studies, through the potentiation of the classical antidepressant treatment [289, 290]. In this way, it was showed, for example, that there is a positive effect generated by adjacent zinc therapy to imipramine medication in the case of treatment resistant depression [291]. In addition, an anxiolytic effect of zinc was recorded in anxiety disorder preclinical [292, 293] and clinical [294] discoveries backing up this hypothesis that could raise hope for the appearance of a new therapeutic avenue for patients fighting with anxiety, comorbid depression, or depression [295].

Moreover, the influence of the ascorbic acid, an element of the antioxidant defense [296], given to an animal model with induced depressive symptoms, pointed out to a fast and serious reverse effect for the behavioural and biochemical deficiencies associated with it [108], therefore proving its potential positive effect in therapy.

Also, lately some studies suggested that omega-3-fatty acids, initially given as adjuvant in psychotropic treatment of depressive disorder [297–299], can be extremely useful in MDD therapy [300]. In this way, new studies regarding the potential of omega-3-fatty acids in walnuts and fish oils were designed in order to overtake these drawbacks and to further investigate this potential therapy direction (as reviewed by [301]).

Other studies showed that lycopene (a carotenoid antioxidant found in tomatoes and other fruits and vegetables [302]) may be an important antioxidant compound, which exhibits no toxic reactions in the animal body [303]. In this way, Francis and Stevenson found that a tomato-rich diet can prevent depressive manifestations and revealed an independent link to lower prevalence rates of depressive behaviours in an elderly community population aged 70 years and over, without any interconnection with intake of other vegetables and depressive symptomatology [304].

In addition, it seems that individual diet and nutrition are highly important to the brain mechanisms involved in mood and affective disorders. In this way, it was showed that a diet saturated in fats and carbohydrates can cause several mood-related brain mechanisms damage [305]. In this way, increased oxidative stress due to unbalanced alimentation was observed in common affective animal models as influencing ROS levels, ROS production, and lipid peroxidation in brain tissues [306]. In addition, a correlation between high carbohydrate diet and cognitive impairments was previously demonstrated [307]. Thus, by adopting a healthy diet, the oxidative stress levels may decrease resulting in a lower mood disorder development and even decreased predisposition. Therefore, further research on this approach may be extremely useful.

Several other studies [308, 309] showed that physical exercises can also be an important component in MDD and ANX treatments. From here an assumption could be made that they might also prevent affective disorders development. Even though the mechanisms underlying the relationship between the effects of exercising and the affective disorders are unknown, some suspect also the possible involvement of oxidative stress [310–313]. However, there are also some controversies about this matter, since some studies supported the protective role of exercise against oxidative stress, while others demonstrated increased oxidative stress markers after acute aerobic [310, 311] and anaerobic exercise [313, 314]. In fact, it seems that ROS produced during exercise sessions provoke specific adaptation and increased regulation in antioxidant endogenous defenses, while antioxidant enzymes activity is leading to higher resistance to oxidative stress [315, 316]. Also, our group previously showed that preadministration of vitamin C, for example, could prevent some oxidative stress manifestations, generated as a result of 40-minute bout of bicycle exercise in young untrained subjects [316]. Thus it seems that the correlation between physical exercise in affective disorders patients and oxidative stress levels may lead to the possibility of employing physical exercise as a potential preventive therapy.

## 7. Conclusions

Due to the high oxygen use and many modulatory systems which rely on redox potential exchange, it seems that the CNS can be excessively exposed to oxidative stress. The correlation between the affective disorders which are a group of well-studied psychiatric disorders sharing common socioaffective features and the almost ubiquitous pathological oxidative stress can be described in a multifactorial background as an important mechanism of central nervous system impairment. Whether the obvious changes which occur in oxidative balance of the mood disorder patients are a part of the constitutive mechanism or a collateral effect yet remains an interesting question. However it is now clear that oxidative stress is a component of these diseases being characterized by different aspects in a disease dependent manner. Therefore, a significant pattern of oxidative stress involvement in affective disorders development can be theorized by further proposing several biological markers that could be assessed in indicating the oxidative status or antioxidant therapy efficiency.

## Figures and Tables

**Table 1:** Main animal models for the affective disorders (adapted from [59–63]).

Induction method	Modelled affective disorders feature	Experiment design	Description
Natural behaviour	Repetitive/stereotypic behaviour [64]	Repetitive behaviour in anxiety assessment tests	Obsessive-compulsive-like behaviour in common anxiety

Drug administration	Bipolar disorder-associated hyperactivity [65]	Locomotor activity evaluation in psychostimulants administration	Psychostimulants can cause hyperactivity
	Drug-induced anxiety [66]	Pentylenetetrazol, sodium lactate, m-chlorophenylpiperazine, cholecystokinin administration	Several drugs can be used to generate anxiogenic responses
	Withdrawal-induced depression [67]	Addictive substances administration	Depression can also occur as a specific symptom of drug withdrawal

Physiological stress	Manic-like behaviour [68]	Locomotor activity, aggressivity, changes in sexual activity during sleep deprivation	Sleep deprivation (>72 h) causes manic-like behaviour
	Hyperthermia induced anxiety [69]	Anxiety assessment in high environmental temperatures	Anxiety-like behaviour can be induced by high environment temperatures
	Helplessness-induced depression [70]	Iterative physiological stress	Animals learn that no escape conditions are provided; therefore they fail to exhibit escape behaviour also in the absence of the stimuli

Psychological stress	Resident-intruder paradigm-based aggressivity [71]	Locomotor activity, aggressivity, changes in sexual activity during social stress	Aggressive behaviour can be a collateral effect in resident-intruder paradigm
	Ultrasonic vocalizations-induced anxiety [72, 73]	Ultrasonic distress in mouse pups separated from their mothers	The decrease in the number of calls, anxiolytic effect
	Hyponeophagia-induced anxiety [74]	Feeding behaviour during/after anxiogenic stimulus of novelty	Novelty in food or environment suppressed feeding
	Maternal deprivation [75, 76]	Maternal deprivation during early postnatal phases	Although controverted, maternal deprivation during infancy can cause depressive disorders occurrence in early adulthood
	Resident-intruder paradigm and social defeat-based depression [77]	Depression assessment in males during consecutive cohabituation	Males can be exposed to psychological stress as a result of consecutive habitation in cages
	Social hierarchization-based depression [78]	Depressive behaviour in tree shrews social hierarchy and subordination	Natural depressive behaviour can occur in different species as a result to social hierarchy

Conflictual stimuli	Vogel-punished drinking [79]	Hydration habits in anxiety	Drinking behaviour is altered when anxiogenic stimuli are applied
	Geller-Seifter task [80]	Feeding behaviour in anxiogenic stimulation	Feeding behaviour is altered when anxiogenic stimuli are applied
	Cognitive Pavlovian [81]	Behavioural changes in Pavlovian conditions	When disagreeable stimuli are applied anxiety behaviour occurs

Neurosurgical model	Olfactory bulbectomy [82]	Behavioural assessment after olfactory bulbectomy	Specific depressive behaviour occurs after olfactory bulb removal

Neurodevelopmental model	Clomipramine administration [83]	Anxiety behaviour in neonatal clomipramine administration	Baby rats exposed to repeated injections of clomipramine develop anxiety-like features in adulthood

Genetic engineering	Selective breeding [84]	Manic behaviour assessment in different strains	Particular strain-specific behavioural features
	Selective breeding [85]	Anxious behaviour during selective breeding	In order to maximize anxious behaviour, the animals are either inbred or outbred
	Single gene manipulation [86]	Anxious phenotyping and single gene manipulation	Knock-out and transgenic mice based on anxiety genes manipulation
	Selective breeding [87, 88]	Depressive behaviour during selective breeding	A strong genetic predisposition to depression can be obtained through high depressive behaviour strains breeding
	Genetic and ontogenetic modelling [89]	Genetic and ontogenetic modelling of depressive traits	Forward or reverse genetic techniques facilitate blockade or stimulation of neuronal activity

**Table 2:** Short overview for the oxidative stress modifications in some affective disorders in animal models.

Followed disease	Animal model/test	Oxidative disturbances
Bipolar disorder	Manic phase, induced with amphetamine	Brain: ↑ SOD production; ↑ TBARS [90] ↑ protein and lipid oxidative damage [91] ↓ SOD: ↓ CAT specific activity [92] ↑ lipid peroxidation [93] ↑ protein aggregation of 4-HNE (a major product of lipid peroxidation) [94]
	Manic phase, chronic amphetamine administration	Submitochondrial fragments of prefrontal cortex and hippocampus: ↑ superoxide production [91] ↓ GSH-Px; ↓ glutathione; ↓ vitamin C [93]
	Manic phase, induced with ouabain	↑ TBARS; ↑ superoxide production; ↑ carbonyl content [95–98] ↑ SOD; ↓ CAT [95]

Depression	Chronic mild stress	↑ superoxide in hippocampus; ↑ TBARS in cortex [99] ↓ antioxidant GST gene expression [100]
	Olfactory bulbectomy model	↓ CAT in blood stream; ↓ GSH; ↓ GSH-Px; ↑ SOD [101]
	Chronic unpredictable mild stress (CUMS)	↑ liver MDA; ↓ TAC (total antioxidant capability); ↓ GSH; ↓ SOD; ↓ CAT [102]
	Swimming restraint	↓ plasma GSH; ↓ plasma TBARS [103]

Anxiety	Fetal alcohol exposure	Hippocampus, cerebellum: ↑ lipid peroxidation; ↑ protein oxidation; ↓ GSH [104]
	Immobilization stress	↑ lipid peroxidation; ↑ nitrite; ↓ GSH; ↓ CAT [105]
	Chronic social isolation	Hepatic levels: ↓ GSH; ↓ glutathione reductase; ↑ CAT; ↑ glutathione S-transferase [106]
	Ovariectomy-induced	Plasma: ↑ 8-isoprostane; hippocampus: ↑ protein carbonylation [107]
	PLTP knock-out model	↓ vitamin E; ↑ oxidative stress markers in phospholipid transfer protein knock-out mice [108]
	Vit. A subacute supplementation	↑ lipid peroxidation; ↑ protein carbonylation; ↑ protein thiol oxidation; SOD and CAT, altered, induced by vitamin A [109]

Posttraumatic stress disorder (PTSD) model	Single prolonged stress	Amygdala: ↓ glutathione reductase; plasma: ↑ 8-isoprostanes levels [110]

Note: SOD: superoxide dismutase; TBARS: thiobarbituric acid reactive substances; CAT: catalase; 4-HNE: 4-hydroxynonenal; MDA: malondialdehyde; 4-HDA: 4-hydroxyalkenals; GSH: glutathione; GHS-Px: glutathione peroxidase; TAC: total antioxidant capacity; GST: glutathione-S-transferase.

## References

[1] Halliwell B. (2012). Free radicals and antioxidants: updating a personal view. *Nutrition Reviews*.

[2] Hirth F. (2011). *Drosophila melanogaster* in the study of human neurodegeneration. *CNS and Neurological Disorders—Drug Targets*.

[3] Rouault T. A. (2013). Iron metabolism in the CNS: implications for neurodegenerative diseases. *Nature Reviews Neuroscience*.

[4] Ghavami S., Shojaei S., Yeganeh B. (2014). Autophagy and apoptosis dysfunction in neurodegenerative disorders. *Progress in Neurobiology*.

[5] Hudson J. I., Pope H. G. (1990). Affective spectrum disorder: does antidepressant response identify a family of disorders with a common pathophysiology?. *The American Journal of Psychiatry*.

[6] Hudson J. I., Mangweth B., Pope H. G. (2003). Family study of affective spectrum disorder. *Archives of General Psychiatry*.

[7] Michel T. M., Pülschen D., Thome J. (2012). The role of oxidative stress in depressive disorders. *Current Pharmaceutical Design*.

[8] Black C. N., Bot M., Scheffer P. G., Cuijpers P., Penninx B. W. J. H. (2015). Is depression associated with increased oxidative stress? A systematic review and meta-analysis. *Psychoneuroendocrinology*.

[9] Vaváková M., Ďuračková Z., Trebatická J. (2015). Markers of oxidative stress and neuroprogression in depression disorder. *Oxidative Medicine and Cell Longevity*.

[10] Bouayed J., Rammal H., Soulimani R. (2009). Oxidative stress and anxiety. Relationship and cellular pathways. *Oxidative Medicine and Cellular Longevity*.

[11] Smaga I., Niedzielska E., Gawlik M. (2015). Oxidative stress as an etiological factor and a potential treatment target of psychiatric disorders. Part 2. Depression, anxiety, schizophrenia and autism. *Pharmacological Reports*.

[12] Andreazza A. C., Kauer-Sant'Anna M., Frey B. N. (2008). Oxidative stress markers in bipolar disorder: a meta-analysis. *Journal of Affective Disorders*.

[13] Ng F., Berk M., Dean O., Bush A. I. (2008). Oxidative stress in psychiatric disorders: evidence base and therapeutic implications. *International Journal of Neuropsychopharmacology*.

[14] Berk M., Kapczinski F., Andreazza A. C. (2011). Pathways underlying neuroprogression in bipolar disorder: focus on inflammation, oxidative stress and neurotrophic factors. *Neuroscience and Biobehavioral Reviews*.

[15] Ersoy M. A., Selek S., Celik H. (2008). Role of oxidative and antioxidative parameters in etiopathogenesis and prognosis of panic disorder. *International Journal of Neuroscience*.

[16] Gul I. G., Karlidag R., Cumurcu B. E. (2013). The effect of agoraphobia on oxidative stress in panic disorder. *Psychiatry Investigation*.

[17] Kandemir H., Abuhandan M., Aksoy N., Savik E., Kaya C. (2013). Oxidative imbalance in child and adolescent patients with obsessive compulsive disorder. *Journal of Psychiatric Research*.

[18] Padurariu M., Ciobica A., Hritcu L., Stoica B., Bild W., Stefanescu C. (2010). Changes of some oxidative stress markers in the serum of patients with mild cognitive impairment and Alzheimer's disease. *Neuroscience Letters*.

[19] Stefanescu C., Ciobica A. (2012). The relevance of oxidative stress status in first episode and recurrent depression. *Journal of Affective Disorders*.

[20] Ciobica A., Padurariu M., Dobrin I., Stefanescu C., Dobrin R. (2011). Oxidative stress in schizophrenia—focusing on the main markers. *Psychiatria Danubina*.

[21] Padurariu M., Ciobica A., Lefter R., Serban I. L., Stefanescu C., Chirita R. (2013). The oxidative stress hypothesis in Alzheimer's disease. *Psychiatria Danubina*.

[22] Bild W., Ciobica A. (2013). Angiotensin-(1–7) central administration induces anxiolytic-like effects in elevated plus maze and decreased oxidative stress in the amygdala. *Journal of Affective Disorders*.

[23] Ciobica A., Hritcu L., Padurariu M., Dobrin R., Bild V. (2010). Effects of serotonin depletion on behavior and neuronal oxidative stress status in rat: relevance for anxiety and affective disorders. *Advances in Medical Sciences*.

[24] Hritcu L., Ciobica A. (2013). Intranigral lipopolysaccharide administration induced behavioral deficits and oxidative stress damage in laboratory rats: relevance for Parkinson's disease. *Behavioural Brain Research*.

[25] Hritcu L., Ciobica A., Stefan M., Mihasan M., Palamiuc L., Nabeshima T. (2011). Spatial memory deficits and oxidative stress damage following exposure to lipopolysaccharide in a rodent model of Parkinson's disease. *Neuroscience Research*.

[26] Bild W., Ciobica A., Padurariu M., Bild V. (2013). The interdependence of the reactive species of oxygen, nitrogen, and carbon. *Journal of Physiology and Biochemistry*.

[27] Massaad C. A., Klann E. (2011). Reactive oxygen species in the regulation of synaptic plasticity and memory. *Antioxidants and Redox Signaling*.

[28] Halliwell B. (2011). Free radicals and antioxidants—quo vadis?. *Trends in Pharmacological Sciences*.

[29] Lehtinen M. K., Bonni A. (2006). Modeling oxidative stress in the central nervous system. *Current Molecular Medicine*.

[30] Maes M., Yirmyia R., Noraberg J. (2009). The inflammatory & neurodegenerative (I&ND) hypothesis of depression: leads for future research and new drug developments in depression. *Metabolic Brain Disease*.

[31] Shao L., Martin M. V., Watson S. J. (2008). Mitochondrial involvement in psychiatric disorders. *Annals of Medicine*.

[32] Clay H. B., Sillivan S., Konradi C. (2011). Mitochondrial dysfunction and pathology in bipolar disorder and schizophrenia. *International Journal of Developmental Neuroscience*.

[33] Hovatta I., Juhila J., Donner J. (2010). Oxidative stress in anxiety and comorbid disorders. *Neuroscience Research*.

[34] Kumar K., Sharma S., Kumar P., Deshmukh R. (2013). Therapeutic potential of GABA_B_ receptor ligands in drug addiction, anxiety, depression and other CNS disorders. *Pharmacology Biochemistry and Behavior*.

[35] Ciranna L. (2006). Serotonin as a modulator of glutamate- and GABA-mediated neurotransmission: implications in physiological functions and in pathology. *Current Neuropharmacology*.

[36] Engin E., Treit D. (2007). The role of hippocampus in anxiety: intracerebral infusion studies. *Behavioural Pharmacology*.

[37] Steimer T. (2002). The biology of fear- and anxiety-related behaviors. *Dialogues in Clinical Neuroscience*.

[38] Distler M. G., Palmer A. A. (2012). Role of glyoxalase 1 (Glo1) and methylglyoxal (MG) in behavior: recent advances and mechanistic insights. *Frontiers in Genetics*.

[39] Hovatta I., Tennant R. S., Helton R. (2005). Glyoxalase 1 and glutathione reductase 1 regulate anxiety in mice. *Nature*.

[40] Hassan W., Silva C. E. B., Mohammadzai I. U., da Rocha J. B. T., Landeira-Fernandez J. (2014). Association of oxidative stress to the genesis of anxiety: implications for possible therapeutic interventions. *Current Neuropharmacology*.

[41] Masood A., Nadeem A., Mustafa S. J., O'Donnell J. M. (2008). Reversal of oxidative stress-induced anxiety by inhibition of phosphodiesterase-2 in mice. *Journal of Pharmacology and Experimental Therapeutics*.

[42] Salim S. (2014). Oxidative stress and psychological disorders. *Current Neuropharmacology*.

[43] Cengiz M., Bayoglu B., Alansal N. O., Cengiz S., Dirican A., Kocabasoglu N. (2015). Pro198Leu polymorphism in the oxidative stress gene, glutathione peroxidase-1, is associated with a gender-specific risk for panic disorder. *International Journal of Psychiatry in Clinical Practice*.

[44] Liu F., Havens J., Yu Q. (2014). The link between angiotensin II-mediated anxiety and mood disorders with NADPH oxidase-induced oxidative stress. *International Journal of Physiology, Pathophysiology and Pharmacology*.

[45] Orhan N., Kucukali C. I., Cakir U., Seker N., Aydin M. (2012). Genetic variants in nuclear-encoded mitochondrial proteins are associated with oxidative stress in obsessive compulsive disorders. *Journal of Psychiatric Research*.

[46] Pagano G., Talamanca A. A., Castello G. (2014). Oxidative stress and mitochondrial dysfunction across broad-ranging pathologies: toward mitochondria-targeted clinical strategies. *Oxidative Medicine and Cellular Longevity*.

[47] Deschwanden A., Karolewicz B., Feyissa A. M. (2011). Reduced metabotropic glutamate receptor 5 density in major depression determined by [^11^C]ABP688 PET and postmortem study. *The American Journal of Psychiatry*.

[48] Petroff O. A. C. (2002). GABA and glutamate in the human brain. *The Neuroscientist*.

[49] Hasler G., van der Veen J. W., Tumonis T., Meyers N., Shen J., Drevets W. C. (2007). Reduced prefrontal glutamate/glutamine and *γ*-aminobutyric acid levels in major depression determined using proton magnetic resonance spectroscopy. *Archives of General Psychiatry*.

[50] Eser D., Schüle C., Baghai T. C., Romeo E., Rupprecht R. (2007). Neuroactive steroids in depression and anxiety disorders: clinical studies. *Neuroendocrinology*.

[51] Sah R., Galeffi F., Ahrens R., Jordan G., Schwartz-Bloom R. D. (2002). Modulation of the GABA_A_-gated chloride channel by reactive oxygen species. *Journal of Neurochemistry*.

[52] Wang M., Perova Z., Arenkiel B. R., Li B. (2014). Synaptic modifications in the medial prefrontal cortex in susceptibility and resilience to stress. *The Journal of Neuroscience*.

[53] Li B., Piriz J., Mirrione M. (2011). Synaptic potentiation onto habenula neurons in the learned helplessness model of depression. *Nature*.

[54] Sin Y. M., Teh W. F., Wong M. K., Reddy P. K. (1990). Effect of mercury on glutathione and thyroid hormones. *Bulletin of Environmental Contamination and Toxicology*.

[55] Aoyama K., Matsubara K., Fujikawa Y. (2000). Nitration of manganese superoxide dismutase in cerebrospinal fluids is a marker for peroxynitrite-mediated oxidative stress in neurodegenerative diseases. *Annals of Neurology*.

[56] Herken H., Gurel A., Selek S. (2007). Adenosine deaminase, nitric oxide, superoxide dismutase, and xanthine oxidase in patients with major depression: impact of antidepressant treatment. *Archives of Medical Research*.

[57] Ozdemir P. G., Kaplan I., Uysal C. (2015). Serum total oxidant and antioxidant status in earthquake survivors with post-traumatic stress disorder. *Acta Neuropsychiatrica*.

[58] Lang U. E., Borgwardt S. (2013). Molecular mechanisms of depression: perspectives on new treatment strategies. *Cellular Physiology and Biochemistry*.

[59] Czéh B., Fuchs E., Wiborg O., Simon M. (2016). Animal models of major depression and their clinical implications. *Progress in Neuro-Psychopharmacology and Biological Psychiatry*.

[60] Crabbe J. C., Wahlsten D., Dudek B. C. (1999). Genetics of mouse behavior: interactions with laboratory environment. *Science*.

[61] Slattery D. A., Cryan J. F., Cryan J. F., Leonard B. E. (2011). Animal models of depression—where are we going?. *Depression: From Psychopathology to Pharmacotherapy*.

[62] Cryan J. F., Slattery D. A. (2007). Animal models of mood disorders: recent developments. *Current Opinion in Psychiatry*.

[63] Slattery D. A., Cryan J. F. (2014). The ups and downs of modelling mood disorders in rodents. *ILAR Journal*.

[64] Winslow J. T., Insel T. R. (1991). Social status in pairs of male squirrel monkeys determines the behavioral response to central oxytocin administration. *Journal of Neuroscience*.

[65] Herman L., Hougland T., El-Mallakh R. S. (2007). Mimicking human bipolar ion dysregulation models mania in rats. *Neuroscience and Biobehavioral Reviews*.

[66] Shekhar A., Keim S. R., Simon J. R., McBride W. J. (1996). Dorsomedial hypothalamic GABA dysfunction produces physiological arousal following sodium lactate infusions. *Pharmacology Biochemistry and Behavior*.

[67] Barr A. M., Markou A., Phillips A. G. (2002). A ‘crash’ course on psychostimulant withdrawal as a model of depression. *Trends in Pharmacological Sciences*.

[68] Szabo S. T., Machado-Vieira R., Yuan P. (2009). Glutamate receptors as targets of protein kinase C in the pathophysiology and treatment of animal models of Mania. *Neuropharmacology*.

[69] van Bogaert M. J. V., Groenink L., Oosting R. S., Westphal K. G. C., Van Der Gugten J., Olivier B. (2006). Mouse strain differences in autonomic responses to stress. *Genes, Brain and Behavior*.

[70] Maier S. F., Seligman M. E. P. (1976). Learned helplessness: theory and evidence. *Journal of Experimental Psychology: General*.

[71] Einat H. (2007). Establishment of a battery of simple models for facets of bipolar disorder: a practical approach to achieve increased validity, better screening and possible insights into endophenotypes of disease. *Behavior Genetics*.

[72] Nastiti K., Benton D., Brain P. F., Haug M. (1991). The effects of 5-HT receptor ligands on ultrasonic calling in mouse pups. *Neuroscience and Biobehavioral Reviews*.

[73] Nastiti K., Benton D., Brain P. (1991). The effects of compounds acting at the benzodiazepine receptor complex on the ultrasonic calling of mouse pups. *Behavioural Pharmacology*.

[74] Bodnoff S. R., Suranyi-Cadotte B., Aitken D. H., Quirion R., Meaney M. J. (1988). The effects of chronic antidepressant treatment in an animal model of anxiety. *Psychopharmacology*.

[75] Pryce C. R., Rüedi-Bettschen D., Dettling A. C., Feldon J. (2002). Early life stress: long-term physiological impact in rodents and primates. *News in Physiological Sciences*.

[76] Pryce C. R., Rüedi-Bettschen D., Dettling A. C. (2005). Long-term effects of early-life environmental manipulations in rodents and primates: potential animal models in depression research. *Neuroscience and Biobehavioral Reviews*.

[77] Kudryavtseva N. N., Bakshtanovskaya I. V., Koryakina L. A. (1991). Social model of depression in mice of C57BL/6J strain. *Pharmacology, Biochemistry and Behavior*.

[78] Fuchs E. (2005). Social stress in tree shrews as an animal model of depression: an example of a behavioral model of a CNS disorder. *CNS Spectrums*.

[79] Vogel J. R., Beer B., Clody D. E. (1971). A simple and reliable conflict procedure for testing anti-anxiety agents. *Psychopharmacologia*.

[80] Geller I., Seifter J. (1960). The effects of meprobamate, barbiturates, d-amphetamine and promazine on experimentally induced conflict in the rat. *Psychopharmacologia*.

[81] Ervin G. N., Cooper B. R. (1988). Use of conditioned taste aversion as a conflict model: effects of anxiolytic drugs. *Journal of Pharmacology and Experimental Therapeutics*.

[82] Kelly J. P., Wrynn A. S., Leonard B. E. (1997). The olfactory bulbectomized rat as a model of depression: an update. *Pharmacology and Therapeutics*.

[83] Andersen S. L., Greene-Colozzi E. A., Sonntag K. C. (2010). A novel, multiple symptom model of obsessive-compulsive-like behaviors in animals. *Biological Psychiatry*.

[84] Einat H. (2007). Different behaviors and different strains: potential new ways to model bipolar disorder. *Neuroscience and Biobehavioral Reviews*.

[85] Crawley J. N., Davis L. G. (1982). Baseline exploratory activity predicts anxiolytic responsiveness to diazepam in five mouse strains. *Brain Research Bulletin*.

[86] Heinrichs S. C., Min H., Tamraz S., Carmouché M., Boehme S. A., Vale W. W. (1997). Anti-sexual and anxiogenic behavioral consequences of corticotropin-releasing factor overexpression are centrally mediated. *Psychoneuroendocrinology*.

[87] Brunelli S. A., Hofer M. A. (2007). Selective breeding for infant rat separation-induced ultrasonic vocalizations: developmental precursors of passive and active coping styles. *Behavioural Brain Research*.

[88] Sartorius A., Mahlstedt M. M., Vollmayr B., Henn F. A., Ende G. (2007). Elevated spectroscopic glutamate/*γ*-amino butyric acid in rats bred for learned helplessness. *NeuroReport*.

[89] Branda C. S., Dymecki S. M. (2004). Talking about a revolution: the impact of site-specific recombinases on genetic analyses in mice. *Developmental Cell*.

[90] Frey B. N., Martins M. R., Petronilho F. C., Dal-Pizzol F., Quevedo J., Kapczinski F. (2006). Increased oxidative stress after repeated amphetamine exposure: possible relevance as a model of mania. *Bipolar Disorders*.

[91] Frey B. N., Valvassori S. S., Réus G. Z. (2006). Changes in antioxidant defense enzymes after d-amphetamine exposure: implications as an animal model of mania. *Neurochemical Research*.

[92] Frey B. N., Valvassori S. S., Gomes K. M. (2006). Increased oxidative stress in submitochondrial particles after chronic amphetamine exposure. *Brain Research*.

[93] Andreazza A. C., Kauer-Sant'Anna M., Frey B. N. (2008). Effects of mood stabilizers on DNA damage in an animal model of mania. *Journal of Psychiatry and Neuroscience*.

[94] Tan H., Young L. T., Shao L., Che Y., Honer W. G., Wang J.-F. (2012). Mood stabilizer lithium inhibits amphetamine-increased 4-hydroxynonenal-protein adducts in rat frontal cortex. *International Journal of Neuropsychopharmacology*.

[95] Valvassori S. S., Dal-Pont G. C., Steckert A. V. (2015). Sodium butyrate has an antimanic effect and protects the brain against oxidative stress in an animal model of mania induced by ouabain. *Psychiatry Research*.

[96] Jornada L. K., Valvassori S. S., Steckert A. V. (2011). Lithium and valproate modulate antioxidant enzymes and prevent ouabain-induced oxidative damage in an animal model of mania. *Journal of Psychiatric Research*.

[97] Riegel R. E., Valvassori S. S., Elias G. (2009). Animal model of mania induced by ouabain: evidence of oxidative stress in submitochondrial particles of the rat brain. *Neurochemistry International*.

[98] Riegel R. E., Valvassori S. S., Moretti M. (2010). Intracerebroventricular ouabain administration induces oxidative stress in the rat brain. *International Journal of Developmental Neuroscience*.

[99] Ejchel-Cohen T. F., Wood G. E., Wang J.-F. (2006). Chronic restraint stress decreases the expression of glutathione S-transferase pi2 in the mouse hippocampus. *Brain Research*.

[100] Song C., Killeen A. A., Leonard B. E. (1994). Catalase, superoxide dismutase and glutathione peroxidase activity in neutrophils of sham-operated and olfactory-bulbectomised rats following chronic treatment with desipramine and lithium chloride. *Neuropsychobiology*.

[101] Zhang D., Wen X.-S., Wang X.-Y., Shi M., Zhao Y. (2009). Antidepressant effect of Shudihuang on mice exposed to unpredictable chronic mild stress. *Journal of Ethnopharmacology*.

[102] de Souza F. G., Rodrigues M. D. B., Tufik S., Nobrega J. N., D'Almeida V. (2006). Acute stressor-selective effects on homocysteine metabolism and oxidative stress parameters in female rats. *Pharmacology Biochemistry and Behavior*.

[103] Todorović N., Tomanović N., Gass P., Filipović D. (2016). Olanzapine modulation of hepatic oxidative stress and inflammation in socially isolated rats. *European Journal of Pharmaceutical Sciences*.

[104] Brocardo P. S., Boehme F., Patten A., Cox A., Gil-Mohapel J., Christie B. R. (2012). Anxiety- and depression-like behaviors are accompanied by an increase in oxidative stress in a rat model of fetal alcohol spectrum disorders: protective effects of voluntary physical exercise. *Neuropharmacology*.

[105] Kumar A., Kaur G., Rinwa P. (2014). Buspirone along with melatonin attenuates oxidative damage and anxiety-like behavior in a mouse model of immobilization stress. *Chinese Journal of Natural Medicines*.

[106] Zorumski C. F., Rubin E. H. (2010). *Demystifying Psychiatry: A Resource for Patients and Families*.

[107] Patki G., Allam F. H., Atrooz F. (2013). Grape powder intake prevents ovariectomy-induced anxiety-like behavior, memory impairment and high blood pressure in female wistar rats. *PLoS ONE*.

[108] Desrumaux C., Risold P.-Y., Schroeder H. (2005). Phospholipid transfer protein (PLTP) deficiency reduces brain vitamin E content and increases anxiety in mice. *The FASEB Journal*.

[109] de Oliveira M. R., Silvestrin R. B., Mello e Souza T., Moreira J. C. F. (2007). Oxidative stress in the hippocampus, anxiety-like behavior and decreased locomotory and exploratory activity of adult rats: effects of sub acute vitamin A supplementation at therapeutic doses. *NeuroToxicology*.

[110] Solanki N., Alkadhi I., Atrooz F., Patki G., Salim S. (2015). Grape powder prevents cognitive, behavioral, and biochemical impairments in a rat model of posttraumatic stress disorder. *Nutrition Research*.

[111] Barlow D. H. (2005). *Abnormal Psychology: An Integrative Approach*.

[112] Shah N., Eisner T., Farrell M., Raeder C. (1999). An overview of SSRIs for the treatment of depression. *The Journal of the Pharmacy Society of Wisconsin*.

[113] Nutt D. J. (2008). Relationship of neurotransmitters to the symptoms of major depressive disorder. *The Journal of Clinical Psychiatry*.

[114] Krishnan V., Nestler E. J. (2008). The molecular neurobiology of depression. *Nature*.

[115] Behr G. A., Moreira J. C. F., Frey B. N. (2012). Preclinical and clinical evidence of antioxidant effects of antidepressant agents: implications for the pathophysiology of major depressive disorder. *Oxidative Medicine and Cellular Longevity*.

[116] Sarris J., Mischoulon D., Schweitzer I. (2011). Adjunctive nutraceuticals with standard pharmacotherapies in bipolar disorder: a systematic review of clinical trials. *Bipolar Disorders*.

[117] Quevedo J., Reus G., Abelaira H. Tianeptine treatment reverses increase on oxidative damage and decrease of antioxidant defense enzymes into the brain of rats submitted to the chronic mild stress model.

[118] Shalaby A., Kamal S. (2009). Effect of Escitalopram on GABA level and anti-oxidant markers in prefrontal cortex and nucleus accumbens of chronic mild stress exposed albino rats. *International Journal of Physiology, Pathophysiology and Pharmacology*.

[119] Eren I., Naziroglu M., Demirdas A. (2007). Venlafaxine modulates depression-induced oxidative stress in brain and medulla of rat. *Neurochemical Research*.

[120] Tok A., Sener E., Albayrak A. (2012). Effect of mirtazapine on oxidative stress created in rat kidneys by ischemia-reperfusion. *Renal Failure*.

[121] Bilici M., Efe H., Koroglu M. A., Uydu H. A., Bekaroglu M., Deger O. (2001). Antioxidative enzyme activities and lipid peroxidation in major depression: alterations by antidepressant treatments. *Journal of Affective Disorders*.

[122] Gałecki P., Szemraj J., Bieńkiewicz M., Zboralski K., Gałecka E. (2009). Oxidative stress parameters after combined fluoxetine and acetylsalicylic acid therapy in depressive patients. *Human Psychopharmacology*.

[123] Sarandol A., Sarandol E., Eker S. S., Erdinc S., Vatansever E., Kirli S. (2007). Major depressive disorder is accompanied with oxidative stress: short-term antidepressant treatment does not alter oxidative-antioxidative systems. *Human Psychopharmacology*.

[124] Gałecki P., Szemraj J., Bieńkiewicz M., Florkowski A., Gałecka E. (2009). Lipid peroxidation and antioxidant protection in patients during acute depressive episodes and in remission after fluoxetine treatment. *Pharmacological Reports*.

[125] Kim D. H., Li H., Yoo K.-Y., Lee B.-H., Hwang I. K., Won M. H. (2007). Effects of fluoxetine on ischemic cells and expressions in BDNF and some antioxidants in the gerbil hippocampal CA1 region induced by transient ischemia. *Experimental Neurology*.

[126] Ozcan M. E., Gulec M., Ozerol E., Polat R., Akyol O. (2004). Antioxidant enzyme activities and oxidative stress in affective disorders. *International Clinical Psychopharmacology*.

[127] Srivastava N., Barthwal M. K., Dalal P. K. (2002). A study on nitric oxide, *β*-adrenergic receptors and antioxidant status in the polymorphonuclear leukocytes from the patients of depression. *Journal of Affective Disorders*.

[128] Khanzode S. D., Dakhale G. N., Khanzode S. S., Saoji A., Palasodkar R. (2003). Oxidative damage and major depression: the potential antioxidant action of selective serotonin-re-uptake inhibitors. *Redox Report*.

[129] Kodydková J., Vávrová L., Zeman M. (2009). Antioxidative enzymes and increased oxidative stress in depressive women. *Clinical Biochemistry*.

[130] Padurariu M., Ciobica A., Dobrin I., Stefanescu C. (2010). Evaluation of antioxidant enzymes activities and lipid peroxidation in schizophrenic patients treated with typical and atypical antipsychotics. *Neuroscience Letters*.

[131] Palta P., Samuel L. J., Miller E. R., Szanton S. L. (2014). Depression and oxidative stress: results from a meta-analysis of observational studies. *Psychosomatic Medicine*.

[132] Dimopoulos N., Piperi C., Psarra V., Lea R. W., Kalofoutis A. (2008). Increased plasma levels of 8-iso-PGF2*α* and IL-6 in an elderly population with depression. *Psychiatry Research*.

[133] Müller C. P., Reichel M., Mühle C., Rhein C., Gulbins E., Kornhuber J. (2015). Brain membrane lipids in major depression and anxiety disorders. *Biochimica et Biophysica Acta (BBA)—Molecular and Cell Biology of Lipids*.

[134] Wilczyńska A. (2013). Fatty acids in treatment and prevention of depression. *Psychiatria Polska*.

[135] Sublette M. E., Ellis S. P., Geant A. L., Mann J. J. (2011). Meta-analysis of the effects of Eicosapentaenoic Acid (EPA) in clinical trials in depression. *Journal of Clinical Psychiatry*.

[136] Zhao Z., Wang W., Guo H., Zhou D. (2008). Antidepressant-like effect of liquiritin from *Glycyrrhiza uralensis* in chronic variable stress induced depression model rats. *Behavioural Brain Research*.

[137] Posser T., Kaster M. P., Baraúna S. C., Rocha J. B. T., Rodrigues A. L. S., Leal R. B. (2009). Antidepressant-like effect of the organoselenium compound ebselen in mice: evidence for the involvement of the monoaminergic system. *European Journal of Pharmacology*.

[138] Brown A. D. H., Barton D. A., Lambert G. W. (2009). Cardiovascular abnormalities in patients with major depressive disorder: autonomic mechanisms and implications for treatment. *CNS Drugs*.

[139] Maes M., Fišar Z., Medina M., Scapagnini G., Nowak G., Berk M. (2012). New drug targets in depression: inflammatory, cell-mediate immune, oxidative and nitrosative stress, mitochondrial, antioxidant, and neuroprogressive pathways. and new drug candidates-Nrf2 activators and GSK-3 inhibitors. *Inflammopharmacology*.

[140] Maes M. (2008). The cytokine hypothesis of depression: inflammation, oxidative & nitrosative stress (IO&NS) and leaky gut as new targets for adjunctive treatments in depression. *Neuroendocrinology Letters*.

[141] Catena-Dell'Osso M., Bellantuono C., Consoli G., Baroni S., Rotella F., Marazziti D. (2011). Inflammatory and neurodegenerative pathways in depression: a new avenue for antidepressant development?. *Current Medicinal Chemistry*.

[142] Kobrosly R., van Wijngaarden E. (2010). Associations between immunologic, inflammatory, and oxidative stress markers with severity of depressive symptoms: an analysis of the 2005-2006 National Health and Nutrition Examination Survey. *NeuroToxicology*.

[143] Benes F. M., Matzilevich D., Burke R. E., Walsh J. (2006). The expression of proapoptosis genes is increased in bipolar disorder, but not in schizophrenia. *Molecular Psychiatry*.

[144] Hoffman K. L. (2016). *Modeling Neuropsychiatric Disorders in Laboratory Animals*.

[145] Poon H. F., Calabrese V., Scapagnini G., Butterfield D. A. (2004). Free radicals and brain aging. *Clinics in Geriatric Medicine*.

[146] Bazan N. G., Marcheselli V. L., Cole-Edwards K. (2005). Brain response to injury and neurodegeneration: endogenous neuroprotective signaling. *Annals of the New York Academy of Sciences*.

[147] Dröge W., Schipper H. M. (2007). Oxidative stress and aberrant signaling in aging and cognitive decline. *Aging Cell*.

[148] Berk M., Dodd S., Kauer-Sant'anna M. (2007). Dopamine dysregulation syndrome: implications for a dopamine hypothesis of bipolar disorder. *Acta Psychiatrica Scandinavica. Supplementum*.

[149] Grande I., Magalhães P. V., Kunz M., Vieta E., Kapczinski F. (2012). Mediators of allostasis and systemic toxicity in bipolar disorder. *Physiology & Behavior*.

[150] Vieta E., Popovic D., Rosa A. R. (2013). The clinical implications of cognitive impairment and allostatic load in bipolar disorder. *European Psychiatry*.

[151] Kapczinski F., Dias V. V., Kauer-Sant'Anna M. (2009). Clinical implications of a staging model for bipolar disorders. *Expert Review of Neurotherapeutics*.

[152] Shan X., Tashiro H., Lin C.-L. (2003). The identification and characterization of oxidized RNAs in Alzheimer's disease. *Journal of Neuroscience*.

[153] Matarese F., Carrillo-de Santa Pau E., Stunnenberg H. G. (2011). 5-Hydroxymethylcytosine: a new kid on the epigenetic block?. *Molecular Systems Biology*.

[154] Thornalley P. J. (2003). Protecting the genome: defence against nucleotide glycation and emerging role of glyoxalase I overexpression in multidrug resistance in cancer chemotherapy. *Biochemical Society Transactions*.

[155] Krömer S. A., Keßler M. S., Milfay D. (2005). Identification of glyoxalase-I as a protein marker in a mouse model of extremes in trait anxiety. *The Journal of Neuroscience*.

[156] Ditzen C., Jastorff A. M., Kessler M. S. (2006). Protein biomarkers in a mouse model extremes in trait anxiety. *Molecular and Cellular Proteomics*.

[157] Dunlop B. W., Davis P. G. (2008). Combination treatment with benzodiazepines and SSRIs for comorbid anxiety and depression: a review. *Primary Care Companion to the Journal of Clinical Psychiatry*.

[158] Kuloglu M., Atmaca M., Tezcan E., Ustundag B., Bulut S. (2002). Antioxidant enzyme and malondialdehyde levels in patients with panic disorder. *Neuropsychobiology*.

[159] Kuloglu M., Atmaca M., Tezcan E., Gecici Ö., Tunckol H., Ustundag B. (2002). Antioxidant enzyme activities and malondialdehyde levels in patients with obsessive-compulsive disorder. *Neuropsychobiology*.

[160] Valko M., Leibfritz D., Moncol J., Cronin M. T. D., Mazur M., Telser J. (2007). Free radicals and antioxidants in normal physiological functions and human disease. *International Journal of Biochemistry and Cell Biology*.

[161] Delattre J., Beaudeux J. L., Bonnefont-Rousselot D. (2005). *Radicaux Libres et Stress Oxydant. Aspects Biologiques et Pathologiques*.

[162] Rotzinger S., Lovejoy D. A., Tan L. A. (2010). Behavioral effects of neuropeptides in rodent models of depression and anxiety. *Peptides*.

[163] Cryan J. F., Sweeney F. F. (2011). The age of anxiety: role of animal models of anxiolytic action in drug discovery. *British Journal of Pharmacology*.

[164] Lefter R., Cojocaru D., Ciobica A., Paulet I. M., Serban I. L., Anton E. (2014). Aspects of animal models for major neuropsychiatric disorders. *Archives of Biological Sciences*.

[165] Dedic N., Walser S. M., Deussing J. M., Uehara T. (2011). Mouse models of depression. *Psychiatric Disorders—Trends and Developments*.

[166] Eren I., Nazıroğlu M., Demirdaş A. (2007). Venlafaxine modulates depression-induced oxidative stress in brain and medulla of rat. *Neurochemical Research*.

[167] Pal S. N., Dandiya P. C. (1994). Glutathione as a cerebral substrate in depressive behavior. *Pharmacology, Biochemistry and Behavior*.

[168] Eren I., Naziroglu M., Demirdas A. (2007). Protective effects of lamotrigine, aripiprazole and escitalopram on depression-induced oxidative stress in rat brain. *Neurochemical Research*.

[169] Lee C. S., Han E. S., Lee W. B. (2003). Antioxidant effect of phenelzine on MPP^+^-induced cell viability loss in differentiated PC12 cells. *Neurochemical Research*.

[170] Abdel-Wahab B. A., Salama R. H. (2011). Venlafaxine protects against stress-induced oxidative DNA damage in hippocampus during antidepressant testing in mice. *Pharmacology Biochemistry and Behavior*.

[171] Padayatty S. J., Katz A., Wang Y. (2003). Vitamin C as an antioxidant: evaluation of its role in disease prevention. *Journal of the American College of Nutrition*.

[172] Moretti M., Colla A., de Oliveira Balen G. (2012). Ascorbic acid treatment, similarly to fluoxetine, reverses depressive-like behavior and brain oxidative damage induced by chronic unpredictable stress. *Journal of Psychiatric Research*.

[173] Patki G., Solanki N., Atrooz F., Allam F., Salim S. (2013). Depression, anxiety-like behavior and memory impairment are associated with increased oxidative stress and inflammation in a rat model of social stress. *Brain Research*.

[174] Ghio L., Natta W., Rossi P. (2011). Combined venlafaxine and olanzapine prescription in women with psychotic major depression: a case series. *Case Reports in Medicine*.

[175] Todorović N., Tomanović N., Gass P., Filipović D. (2016). Olanzapine modulation of hepatic oxidative stress and inflammation in socially isolated rats. *European Journal of Pharmaceutical Sciences*.

[176] Jeding I., Evans P. J., Akanmu D. (1995). Characterization of the potential antioxidant and pro-oxidant actions of some neuroleptic drugs. *Biochemical Pharmacology*.

[177] Parikh V., Khan M. M., Mahadik S. P. (2003). Differential effects of antipsychotics on expression of antioxidant enzymes and membrane lipid peroxidation in rat brain. *Journal of Psychiatric Research*.

[178] Pillai A., Parikh V., Terry A. V., Mahadik S. P. (2007). Long-term antipsychotic treatments and crossover studies in rats: differential effects of typical and atypical agents on the expression of antioxidant enzymes and membrane lipid peroxidation in rat brain. *Journal of Psychiatric Research*.

[179] Wang H., Xu H., Dyck L. E., Li X.-M. (2005). Olanzapine and quetiapine protect PC12 cells from *β*-amyloid peptide 25–35-induced oxidative stress and the ensuing apoptosis. *Journal of Neuroscience Research*.

[180] Xu H., Wang H., Zhuang L. (2008). Demonstration of an anti-oxidative stress mechanism of quetiapine: implications for the treatment of Alzheimer's disease. *The FEBS Journal*.

[181] Ciobica A., Bild V., Hritcu L., Padurariu M., Bild W. (2011). Effects of angiotensin II receptor antagonists on anxiety and some oxidative stress markers in rat. *Central European Journal of Medicine*.

[182] Ciobica A., Hritcu L., Nastasa V., Padurariu M., Bild W. (2011). Inhibition of central angiotensin converting enzyme exerts anxiolytic effects by decreasing brain oxidative stress. *Journal of Medical Biochemistry*.

[183] Vignes M., Maurice T., Lanté F. (2006). Anxiolytic properties of green tea polyphenol (−)-epigallocatechin gallate (EGCG). *Brain Research*.

[184] Kolosova N. G., Trofimova N. A., Fursova A. Z. (2006). Opposite effects of antioxidants on anxiety in Wistar and OXYS rats. *Bulletin of Experimental Biology and Medicine*.

[185] Post R. M. (2015). Heading off depressive illness evolution and progression to treatment resistance. *Dialogues in Clinical Neuroscience*.

[186] Craddock N., Forty L. (2006). Genetics of affective (mood) disorders. *European Journal of Human Genetics*.

[187] McGuffin P., Rijsdijk F., Andrew M., Sham P., Katz R., Cardno A. (2003). The heritability of bipolar affective disorder and the genetic relationship to unipolar depression. *Archives of General Psychiatry*.

[188] Kendler K. S., Neale M. C., Kessler R. C., Heath A. C., Eaves L. J. (1993). The lifetime history of major depression in women: reliability of diagnosis and heritability. *Archives of General Psychiatry*.

[189] Eley T. C., Collier D., McGuffin P., McGuffin P., Owen M. J., Gottesman I. I. (2002). Anxiety and eating disorders. *Psychiatric Genetics and Genomics*.

[190] Norrholm S. D., Ressler K. J. (2009). Genetics of anxiety and trauma-related disorders. *Neuroscience*.

[191] Skelton K., Ressler K. J., Norrholm S. D., Jovanovic T., Bradley-Davino B. (2012). PTSD and gene variants: new pathways and new thinking. *Neuropharmacology*.

[192] Galanopoulou A. S. (2008). GABA A receptors in normal development and seizures: friends or foes?. *Current Neuropharmacology*.

[193] Zmijewski J. W., Song L., Harkins L., Cobbs C. S., Jope R. S. (2001). Oxidative stress and heat shock stimulate RGS2 expression in 1321N1 astrocytoma cells. *Archives of Biochemistry and Biophysics*.

[194] Nunn C., Zou M.-X., Sobiesiak A. J., Roy A. A., Kirshenbaum L. A., Chidiac P. (2010). RGS2 inhibits *β*-adrenergic receptor-induced cardiomyocyte hypertrophy. *Cellular Signalling*.

[195] Binder E. B., Bradley R. G., Liu W. (2008). Association of FKBP5 polymorphisms and childhood abuse with risk of posttraumatic stress disorder symptoms in adults. *JAMA*.

[196] Guidotti G., Calabrese F., Anacker C., Racagni G., Pariante C. M., Riva M. A. (2013). Glucocorticoid receptor and fkbp5 expression is altered following exposure to chronic stress: modulation by antidepressant treatment. *Neuropsychopharmacology*.

[197] Ressler K. J., Mercer K. B., Bradley B. (2011). Post-traumatic stress disorder is associated with PACAP and the PAC1 receptor. *Nature*.

[198] Masmoudi-Kouki O., Douiri S., Hamdi Y. (2011). Pituitary adenylate cyclase-activating polypeptide protects astroglial cells against oxidative stress-induced apoptosis. *Journal of Neurochemistry*.

[199] Odaka H., Numakawa T., Adachi N. (2014). Cabergoline, dopamine D2 receptor agonist, prevents neuronal cell death under oxidative stress via reducing excitotoxicity. *PLoS ONE*.

[200] Rosin C., Colombo S., Calver A. A., Bates T. E., Skaper S. D. (2005). Dopamine D2 and D3 receptor agonists limit oligodendrocyte injury caused by glutamate oxidative stress and oxygen/glucose deprivation. *GLIA*.

[201] Sankhwar M. L., Yadav R. S., Shukla R. K. (2016). Monocrotophos induced oxidative stress and alterations in brain dopamine and serotonin receptors in young rats. *Toxicology and Industrial Health*.

[202] Low N. C. P., Cui L., Merikangas K. R. (2008). Specificity of familial transmission of anxiety and comorbid disorders. *Journal of Psychiatric Research*.

[203] Merikangas K. R., Li J. J., Stipelman B. (2009). The familial aggregation of cannabis use disorders. *Addiction*.

[204] Smoller J. W., Finn C. T. (2003). Family, twin, and adoption studies of bipolar disorder. *American Journal of Medical Genetics Part C (Seminaries of Medical Genetics)*.

[205] Na H.-R., Kang E.-H., Lee J.-H., Yu B.-H. (2011). The genetic basis of panic disorder. *Journal of Korean Medical Science*.

[206] Lehman C. L., Brown T. A., Palfai T., Barlow D. H. (2002). The effects of alcohol outcome expectancy on a carbon-dioxide challenge in patients with panic disorder. *Behavior Therapy*.

[207] Mehaney D. A., Darwish H. A., Hegazy R. A. (2014). Analysis of oxidative stress status, catalase and catechol-O-methyltransferase polymorphisms in Egyptian vitiligo patients. *PLoS ONE*.

[208] Colucci R., Dragoni F., Moretti S. (2015). Oxidative stress and immune system in vitiligo and thyroid diseases. *Oxidative Medicine and Cellular Longevity*.

[209] Jönsson E. G., Norton N., Forslund K. (2003). Association between a promoter variant in the monoamine oxidase A gene and schizophrenia. *Schizophrenia Research*.

[210] Deckert J., Catalano M., Syagailo Y. V. (1999). Excess of high activity monoamine oxidase A gene promoter alleles in female patients with panic disorder. *Human Molecular Genetics*.

[211] Hamilton S. P., Slager S. L., Heiman G. A. (2000). No genetic linkage or association between a functional promoter polymorphism in the monoamine oxidase-A gene and panic disorder. *Molecular Psychiatry*.

[212] Vanderhaeghen J. J., Signeau J. C., Gepts W. (1975). New peptide in the vertebrate CNS reacting with antigastrin antibodies. *Nature*.

[213] Huppi K., Siwarski D., Pisegna J. R., Wank S. (1995). Chromosomal localization of the gastric and brain receptors for cholecystokinin (CCKAR and CCKBR) in human and mouse. *Genomics*.

[214] Strug L. J., Suresh R., Fyer A. J. (2010). Panic disorder is associated with the serotonin transporter gene (SLC6A4) but not the promoter region (5-HTTLPR). *Molecular Psychiatry*.

[215] Wendland J. R., Moya P. R., Kruse M. R. (2008). A novel, putative gain-of-function haplotype at SLC6A4 associates with obsessive-compulsive disorder. *Human Molecular Genetics*.

[216] Ravindran L. N., Stein M. B., Sadock B. J., Sadock V. A., Ruiz P. (2009). Anxiety disorders: somatic treatment. *Kaplan and Sadock Comprehensive Textbook of Psychiatry*.

[217] Maron E., Nikopensius T., Kõks S. (2005). Association study of 90 candidate gene polymorphisms in panic disorder. *Psychiatric Genetics*.

[218] Preisig M., Bellivier F., Fenton B. T. (2000). Association between bipolar disorder and monoarnine oxidase a gene polymorphisms: results of a multicenter study. *American Journal of Psychiatry*.

[219] Jones I., Craddock N. (2001). Candidate gene studies of bipolar disorder. *Annals of Medicine*.

[220] Anguelova M., Benkelfat C., Turecki G. (2003). A systematic review of association studies investigating genes coding for serotonin receptors and the serotonin transporter: I. Affective disorders. *Molecular Psychiatry*.

[221] Menazza S., Blaauw B., Tiepolo T. (2010). Oxidative stress by monoamine oxidases is causally involved in myofiber damage in muscular dystrophy. *Human Molecular Genetics*.

[222] Bild W., Hritcu L., Stefanescu C., Ciobica A. (2013). Inhibition of central angiotensin II enhances memory function and reduces oxidative stress status in rat hippocampus. *Progress in Neuro-Psychopharmacology and Biological Psychiatry*.

[223] Green E., Craddock N. (2003). Brain-derived neurotrophic factor as a potential risk locus for bipolar disorder: evidence, limitaions, and implications. *Current Psychiatry Reports*.

[224] Hattori E., Liu C., Badner J. A. (2003). Polymorphisms at the G72/G30 gene locus, on 13q33, are associated with bipolar disorder in two independent pedigree series. *American Journal of Human Genetics*.

[225] Cabungcal J.-H., Steullet P., Morishita H. (2013). Perineuronal nets protect fast-spiking interneurons against oxidative stress. *Proceedings of the National Academy of Sciences of the United States of America*.

[226] Schumacher J., Jamra R. A., Becker T. (2005). Evidence for a relationship between genetic variants at the brain-derived neurotrophic factor (BDNF) locus and major depression. *Biological Psychiatry*.

[227] Surtees P. G., Wainwright N. W. J., Willis-Owen S. A. G. (2007). No association between the BDNF Val66Met polymorphism and mood status in a non-clinical community sample of 7389 older adults. *Journal of Psychiatric Research*.

[228] Hashimoto K. (2010). Brain-derived neurotrophic factor as a biomarker for mood disorders: an historical overview and future directions. *Psychiatry and Clinical Neurosciences*.

[229] Zhang X. Y., Chen D.-C., Tan Y.-L. (2015). The interplay between BDNF and oxidative stress in chronic schizophrenia. *Psychoneuroendocrinology*.

[230] Numakawa T., Richards M., Nakajima S. (2014). The role of brain-derived neurotrophic factor in comorbid depression: possible linkage with steroid hormones, cytokines, and nutrition. *Frontiers in Psychiatry*.

[231] Lohoff F. W. (2010). Overview of the genetics of major depressive disorder. *Current Psychiatry Reports*.

[232] Zill P., Baghai T. C., Zwanzger P. (2004). SNP and haplotype analysis of a novel tryptophan hydroxylase isoform (TPH2) gene provide evidence for association with major depression. *Molecular Psychiatry*.

[233] Zhang X., Gainetdinov R. R., Beaulieu J.-M. (2005). Loss-of-function mutation in tryptophan hydroxylase-2 identified in unipolar major depression. *Neuron*.

[234] Kuhn D. M., Sykes C. E., Geddes T. J., Jaunarajs K. L. E., Bishop C. (2011). Tryptophan hydroxylase 2 aggregates through disulfide cross-linking upon oxidation: Possible link to serotonin deficits and non-motor symptoms in Parkinson's disease. *Journal of Neurochemistry*.

[235] Weng R., Shen S., Burton C. (2016). Lipidomic profiling of tryptophan hydroxylase 2 knockout mice reveals novel lipid biomarkers associated with serotonin deficiency. *Analytical and Bioanalytical Chemistry*.

[236] Lydiard R. B. (2003). The role of GABA in anxiety disorders. *Journal of Clinical Psychiatry*.

[237] Unschuld P. G., Ising M., Specht M. (2009). Polymorphisms in the GAD2 gene-region are associated with susceptibility for unipolar depression and with a risk factor for anxiety disorders. *American Journal of Medical Genetics Part B: Neuropsychiatric Genetics*.

[238] Lamigeon C., Prod'Hon C., De Frias V., Michoudet C., Jacquemont B. (2003). Enhancement of neuronal protection from oxidative stress by glutamic acid decarboxylase delivery with a defective herpes simplex virus vector. *Experimental Neurology*.

[239] Lamigeon C., Bellier J. P., Sacchettoni S., Rujano M., Jacquemont B. (2001). Enhanced neuronal protection from oxidative stress by coculture with glutamic acid decarboxylase-expressing astrocytes. *Journal of Neurochemistry*.

[240] Leygraf A., Hohoff C., Freitag C. (2006). Rgs 2 gene polymorphisms as modulators of anxiety in humans?. *Journal of Neural Transmission*.

[241] Smoller J. W., Paulus M. P., Fagerness J. A. (2008). Influence of RGS2 on anxiety-related temperament, personality, and brain function. *Archives of General Psychiatry*.

[242] Koenen K. C., Amstadter A. B., Ruggiero K. J. (2009). *RGS2* and generalized anxiety disorder in an epidemiologic sample of hurricane-exposed adults. *Depression & Anxiety*.

[243] Endale M., Kim S. D., Lee W. M. (2010). Ischemia induces regulator of G protein signaling 2 (RGS2) protein upregulation and enhances apoptosis in astrocytes. *American Journal of Physiology-Cell Physiology*.

[244] Monroy C. A., Mackie D. I., Roman D. L. (2013). A high throughput screen for RGS proteins using steady state monitoring of free phosphate formation. *PLoS ONE*.

[245] Wu Z., Puigserver P., Andersson U. (1999). Mechanisms controlling mitochondrial biogenesis and respiration through the thermogenic coactivator PGC-1. *The Cell*.

[246] World Health Organization (2008). *The Global Burden of Disease: 2004 Update*.

[247] Nutt D. J., Kessler R. C., Alonso J. (2007). Consensus statement on the benefit to the community of ESEMeD (European study of the epidemiology of mental disorders) survey data on depression and anxiety. *Journal of Clinical Psychiatry*.

[248] Kessler R. C. (2007). The global burden of anxiety and mood disorders: putting the European Study of the Epidemiology of Mental Disorders (ESEMeD) findings into perspective. *Journal of Clinical Psychiatry*.

[249] Kessler R. C., Chiu W. T., Demler O., Merikangas K. R., Walters E. E. (2005). Prevalence, severity, and comorbidity of 12-month DSM-IV disorders in the National Comorbidity Survey Replication. *Archives of General Psychiatry*.

[250] Bakunina N., Pariante C. M., Zunszain P. A. (2015). Immune mechanisms linked to depression via oxidative stress and neuroprogression. *Immunology*.

[251] de Sousa R. T., Zarate C. A., Zanetti M. V. (2014). Oxidative stress in early stage bipolar disorder and the association with response to lithium. *Journal of Psychiatric Research*.

[252] Kiełczykowska M., Pasternak K., Musik I., Wrońska-Tyra J., Hordyjewska A. (2006). The influence of different doses of lithium administered in drinking water on lipid peroxidation and the activity of antioxidant enzymes in rats. *Polish Journal of Environmental Studies*.

[253] Machado-Vieira R., Andreazza A. C., Viale C. I. (2007). Oxidative stress parameters in unmedicated and treated bipolar subjects during initial manic episode: a possible role for lithium antioxidant effects. *Neuroscience Letters*.

[254] Banerjee U., Dasgupta A., Rout J. K., Singh O. P. (2012). Effects of lithium therapy on Na+-K+-ATPase activity and lipid peroxidation in bipolar disorder. *Progress in Neuro-Psychopharmacology and Biological Psychiatry*.

[255] Atmaca M., Tezcan E., Kuloglu M., Ustundag B., Tunckol H. (2004). Antioxidant enzyme and malondialdehyde values in social phobia before and after citalopram treatment. *European Archives of Psychiatry and Clinical Neuroscience*.

[256] Frey B. N., Andreazza A. C., Kunz M. (2007). Increased oxidative stress and DNA damage in bipolar disorder: a twin-case report. *Progress in Neuro-Psychopharmacology and Biological Psychiatry*.

[257] Khairova R., Pawar R., Salvadore G. (2012). Effects of lithium on oxidative stress parameters in healthy subjects. *Molecular Medicine Reports*.

[258] Wang P. S., Walker A. M., Tsuang M. T. (2002). Dopamine antagonists and the development of breast cancer. *Archives of General Psychiatry*.

[259] Cui J., Shao L., Young L. T., Wang J.-F. (2007). Role of glutathione in neuroprotective effects of mood stabilizing drugs lithium and valproate. *Neuroscience*.

[260] Shukla G. S., Hussain T., Chandra S. V. (1987). Possible role of regional superoxide dismutase activity and lipid peroxide levels in cadmium neurotoxicity: in vivo and in vitro studies in growing rats. *Life Sciences*.

[261] Selek S., Savas H. A., Gergerlioglu H. S., Bulbul F., Uz E., Yumru M. (2008). The course of nitric oxide and superoxide dismutase during treatment of bipolar depressive episode. *Journal of Affective Disorders*.

[262] Hwang J., Zheng L. T., Ock J. (2008). Inhibition of glial inflammatory activation and neurotoxicity by tricyclic antidepressants. *Neuropharmacology*.

[263] Hadden J. W., Szentivanyi A. (1990). *Immunopharmacology Reviews*.

[264] Bilici M., Efe H., Köroğlu M. A., Uydu H. A., Bekaroğlu M., Değer O. (2001). Antioxidative enzyme activities and lipid peroxidation in major depression: alterations by antidepressant treatments. *Journal of Affective Disorders*.

[265] Inkielewicz-Stêpniak I. (2011). Impact of fluoxetine on liver damage in rats. *Pharmacological Reports*.

[266] Moreno-Fernández A. M., Cordero M. D., Garrido-Maraver J. (2012). Oral treatment with amitriptyline induces coenzyme Q deficiency and oxidative stress in psychiatric patients. *Journal of Psychiatric Research*.

[267] Bautista-Ferrufino M. R., Cordero M. D., Sánchez-Alcázar J. A. (2011). Amitriptyline induces coenzyme Q deficiency and oxidative damage in mouse lung and liver. *Toxicology Letters*.

[268] Milne G. L., Sanchez S. C., Musiek E. S., Morrow J. D. (2007). Quantification of F_2_-isoprostanes as a biomarker of oxidative stress. *Nature Protocols*.

[269] Chung C. P., Schmidt D., Stein C. M., Morrow J. D., Salomon R. M. (2013). Increased oxidative stress in patients with depression and its relationship to treatment. *Psychiatry Research*.

[270] Maes M. (2008). The cytokine hypothesis of depression: inflammation, oxidative & nitrosative stress (IO&NS) and leaky gut as new targets for adjunctive treatments in depression. *Neuroendocrinology Letters*.

[271] Peet M., Horrobin D. F. (2002). A dose-ranging study of the effects of ethyl-eicosapentaenoate in patients with ongoing depression despite apparently adequate treatment with standard drugs. *Archives of General Psychiatry*.

[272] Carvalho A. F., Macêdo D. S., Goulia P., Hyphantis T. N. (2013). N-Acetylcysteine augmentation to tranylcypromine in treatment-resistant major depression. *Journal of Clinical Psychopharmacology*.

[273] Strawn J. R., Saldaña S. N. (2012). Treatment with adjunctive N-acetylcysteine in an adolescent with selective serotonin reuptake inhibitor-resistant anxiety. *Journal of Child and Adolescent Psychopharmacology*.

[274] Maes M., Galecki P., Chang Y. S., Berk M. (2011). A review on the oxidative and nitrosative stress (O&NS) pathways in major depression and their possible contribution to the (neuro)degenerative processes in that illness. *Progress in Neuro-Psychopharmacology and Biological Psychiatry*.

[275] Ibrahim W., Tousson E., El-Masry T., Arafa N., Akela M. (2012). The effect of folic acid as an antioxidant on the hypothalamic monoamines in experimentally induced hypothyroid rat. *Toxicology and Industrial Health*.

[276] Taylor M. J., Carney S. M., Goodwin G. M., Geddes J. R. (2004). Folate for depressive disorders: systematic review and meta-analysis of randomized controlled trials. *Journal of Psychopharmacology*.

[277] Atkuri K. R., Mantovani J. J., Herzenberg L. A., Herzenberg L. A. (2007). N-Acetylcysteine-a safe antidote for cysteine/glutathione deficiency. *Current Opinion in Pharmacology*.

[278] Odlaug B. L., Grant J. E. (2007). N-acetyl cysteine in the treatment of grooming disorders. *Journal of Clinical Psychopharmacology*.

[279] Berk M., Dean O. M., Cotton S. M. (2014). The efficacy of adjunctive N-acetylcysteine in major depressive disorder: a double-blind, randomized, placebo-controlled trial. *Journal of Clinical Psychiatry*.

[280] Marshall K.-A., Reiter R. J., Poeggeler B., Aruoma O. I., Halliwell B. (1996). Evaluation of the antioxidant activity of melatonin in vitro. *Free Radical Biology and Medicine*.

[281] Quera Salva M. A., Hartley S., Barbot F., Alvarez J. C., Lofaso F., Guilleminault C. (2011). Circadian rhythms, melatonin and depression. *Current Pharmaceutical Design*.

[282] Fornaro M., McCarthy M. J., De Berardis D. (2013). Adjunctive agomelatine therapy in the treatment of acute bipolar II depression: a preliminary open label study. *Neuropsychiatric Disease and Treatment*.

[283] Sapède D., Cau E. (2013). The pineal gland from development to function. *Current Topics in Developmental Biology*.

[284] Maes M., Vandoolaeghe E., Neels H. (1997). Lower serum zinc in major depression is a sensitive marker of treatment resistance and of the immune/ inflammatory response in that illness. *Biological Psychiatry*.

[285] Collins J. F., Klevay L. M. (2011). Copper. *Advances in Nutrition*.

[286] Kodama H., Fujisawa C., Bhadhprasit W. (2012). Inherited copper transport disorders: biochemical mechanisms, diagnosis, and treatment. *Current Drug Metabolism*.

[287] Młyniec K., Gaweł M., Doboszewska U. (2015). Essential elements in depression and anxiety. Part II. *Pharmacological Reports*.

[288] Swardfager W., Herrmann N., McIntyre R. S. (2013). Potential roles of zinc in the pathophysiology and treatment of major depressive disorder. *Neuroscience & Biobehavioral Reviews*.

[289] Lai J., Moxey A., Nowak G., Vashum K., Bailey K., McEvoy M. (2012). The efficacy of zinc supplementation in depression: systematic review of randomised controlled trials. *Journal of Affective Disorders*.

[290] Siwek M., Dudek D., Paul I. A. (2009). Zinc supplementation augments efficacy of imipramine in treatment resistant patients: a double blind, placebo-controlled study. *Journal of Affective Disorders*.

[291] Szewczyk B. (2013). Zinc homeostasis and neurodegenerative disorders. *Frontiers in Aging Neuroscience*.

[292] Szewczyk B., Kubera M., Nowak G. (2011). The role of zinc in neurodegenerative inflammatory pathways in depression. *Progress in Neuro-Psychopharmacology & Biological Psychiatry*.

[293] Sayyah M., Olapour A., Saeedabad Y. S., Yazdan Parast R., Malayeri A. (2012). Evaluation of oral zinc sulfate effect on obsessive-compulsive disorder: a randomized placebo-controlled clinical trial. *Nutrition*.

[294] Nowak G. (2015). Zinc, future mono/adjunctive therapy for depression: mechanisms of antidepressant action. *Pharmacological Reports*.

[295] Padayatty S. J., Katz A., Wang Y. (2003). Vitamin C as an antioxidant: evaluation of its role in disease prevention. *The Journal of the American College of Nutrition*.

[296] Kraguljac N. V., Montori V. M., Pavuluri M., Chai H. S., Wilson B. S., Unal S. S. (2009). Efficacy of omega-3 fatty acids in mood disorders—a systematic review and metaanalysis. *Psychopharmacology Bulletin*.

[297] Montgomery P., Richardson A. J. (2008). Omega-3 fatty acids for bipolar disorder. *Cochrane Database of Systematic Reviews*.

[298] Sarris J., Mischoulon D., Schweitzer I. (2012). Omega-3 for bipolar disorder: meta-analyses of use in mania and bipolar depression. *Journal of Clinical Psychiatry*.

[299] Lin P. Y., Su K. P. (2007). A meta-analytic review of double-blind, placebo-controlled trials of antidepressant efficacy of omega-3 fatty acids. *Journal of Clinical Psychiatry*.

[300] Lane K., Derbyshire E., Li W., Brennan C. (2014). Bioavailability and potential uses of vegetarian sources of omega-3 fatty acids: a review of the literature. *Critical Reviews in Food Science and Nutrition*.

[301] Islamian J. P., Mehrali H. (2015). Lycopene as a carotenoid provides radioprotectant and antioxidant effects by quenching radiation-induced free radical singlet oxygen: an overview. *Cell Journal*.

[302] Heber D., Lu Q.-Y. (2002). Overview of mechanisms of action of lycopene. *Experimental Biology and Medicine*.

[303] Niu K., Guo H., Kakizaki M. (2013). A tomato-rich diet is related to depressive symptoms among an elderly population aged 70 years and over: a population-based, cross-sectional analysis. *Journal of Affective Disorders*.

[304] Francis H., Stevenson R. (2013). The longer-term impacts of Western diet on human cognition and the brain. *Appetite*.

[305] Nishikawa T., Edelstein D., Du X. L. (2000). Normalizing mitochondrial superoxide production blocks three pathways of hyperglycaemic damage. *Nature*.

[306] White C. L., Pistell P. J., Purpera M. N. (2009). Effects of high fat diet on Morris maze performance, oxidative stress, and inflammation in rats: contributions of maternal diet. *Neurobiology of Disease*.

[307] Ströhle A. (2009). Physical activity, exercise, depression and anxiety disorders. *Journal of Neural Transmission*.

[308] Ng F., Dodd S., Berk M. (2007). The effects of physical activity in the acute treatment of bipolar disorder: a pilot study. *Journal of Affective Disorders*.

[309] Benitez-Sillero J. D., Perez-Navero J. L., Tasset I., Guillen-Del Castillo M., Gil-Campos M., Tunez I. (2011). Cardiorespiratory fitness and oxidative stress: effect of acute maximal aerobic exercise in children and adolescents. *Journal of Sports Medicine and Physical Fitness*.

[310] Bloomer R. J. (2008). Effect of exercise on oxidative stress biomarkers. *Advances in Clinical Chemistry*.

[311] Pittaluga M., Parisi P., Sabatini S. (2006). Cellular and biochemical parameters of exercise-induced oxidative stress: relationship with training levels. *Free Radical Research*.

[312] Eyre H., Baune B. T. (2012). Neuroimmunological effects of physical exercise in depression. *Brain, Behavior, and Immunity*.

[313] Bloomer R. J., Goldfarb A. H. (2004). Anaerobic exercise and oxidative stress: a review. *Canadian Journal of Applied Physiology*.

[314] Radak Z., Chung H. Y., Goto S. (2008). Systemic adaptation to oxidative challenge induced by regular exercise. *Free Radical Biology and Medicine*.

[315] Lopresti A. L., Hood S. D., Drummond P. D. (2013). A review of lifestyle factors that contribute to important pathways associated with major depression: diet, sleep and exercise. *Journal of Affective Disorders*.

[316] Trofin F.-P., Chirazi M., Honceriu C. (2014). Pre-administration of vitamin C reduces exercise-induced oxidative stress in untrained subjects. *Archives of Biological Sciences*.

